# Distinct lncRNA transcriptional fingerprints characterize progressive stages of multiple myeloma

**DOI:** 10.18632/oncotarget.7442

**Published:** 2016-02-17

**Authors:** Domenica Ronchetti, Luca Agnelli, Elisa Taiana, Serena Galletti, Martina Manzoni, Katia Todoerti, Pellegrino Musto, Francesco Strozzi, Antonino Neri

**Affiliations:** ^1^ Department of Oncology and Hemato-Oncology, University of Milano, Milan, Italy; ^2^ Hematology Unit, Fondazione IRCCS Ca’ Granda, Ospedale Maggiore Policlinico, Milan, Italy; ^3^ Laboratory of Pre-Clinical and Translational Research, IRCCS-CROB, Referral Cancer Center of Basilicata, Rionero in Vulture, Potenza, Italy; ^4^ Bioinformatics Core Facility, Parco Tecnologico Padano, Lodi, Italy

**Keywords:** lncRNA, multiple myeloma, plasma cell dyscrasia, MALAT1, expression profiling

## Abstract

Although many efforts have recently contributed to improve our knowledge of molecular pathogenesis of multiple myeloma (MM), the role and significance of long non-coding RNAs (lncRNAs) in plasma cells (PC) malignancies remains virtually absent. To this aim, we developed a custom annotation pipeline of microarray data investigating lncRNA expression in PCs from 20 monoclonal gammopathies of undetermined significance, 33 smoldering MM, 170 MM, and 36 extra-medullary MMs/plasma cell leukemia patients, and 9 healthy donors. Our study identified 31 lncRNAs deregulated in tumor samples compared to normal controls; among these, the upregulation of MALAT1 appeared associated in MM patients with molecular pathways involving cell cycle regulation, p53-mediated DNA damage response, and mRNA maturation processes. Furthermore, we found 21 lncRNAs whose expression were progressively deregulated trough the more aggressive stages of PC dyscrasia, suggesting a possible role in the progression of the disease. Finally, in the context of molecular heterogeneity of MM, we identified a transcriptional fingerprint in hyperdiploid patients, characterized by the upregulation of lncRNAs/pseudogenes related to ribosomal protein genes, known to be upregulated in this molecular group. Overall, the data provides an important resource for future studies on the functions of lncRNAs in the pathology.

## INTRODUCTION

Multiple myeloma (MM) is a malignant proliferation of antibody-secreting bone marrow plasma cells (PCs) characterized by a wide clinical spectrum ranging from the presumed pre-malignant condition called monoclonal gammopathy of undetermined significance (MGUS), to smoldering MM (SMM), truly overt and symptomatic MM, and extra-medullary myeloma/plasma cell leukemia (PCL) [1–3]. Despite the remarkable improvements in treatment and patient care [4], MM remains an incurable disease.

During the years that have followed human genome sequencing, it has become evident that over 90% of the genome is actively transcribed [5, 6], the majority of transcripts being represented by non-coding RNA (ncRNA) and therefore not translated into proteins. NcRNAs are broadly divided into short (<200 nt) and long (>200 nt) transcripts. Dysregulation of short ncRNAs, particularly miRNAs, has been reported to occur virtually in all types of cancer, including MM, highlighting the usefulness of miRNA profiling in diagnosis, prognosis, and in predicting response to therapy [7, 8]. Notably, miRNAs are currently considered both emerging therapeutic targets and innovative intervention tools in cancer including MM [9–11].

Long non-coding RNAs (lncRNAs) are a very heterogeneous group that lack mRNA properties, although they exhibit a structure and biogenesis that does not differ greatly from mRNAs: indeed, they can be polyadenylated and may operate in either nuclear and/or cytoplasmic fractions. LncRNAs represent more than half of the mammalian noncoding transcriptome and are involved in many biological processes, such as transcriptional gene regulation, maintenance of genomic integrity, X-chromosome inactivation, genomic imprinting, cell differentiation, and development [12]. Among lncRNAs, there are also pseudogenes that have lost their protein-coding ability or are otherwise no longer translated; however, they may be functional, similar to other kinds of non-coding RNA, and can have a regulatory role [13]. LncRNAs can also be classified based on their location relative to nearby protein-coding genes. They originate from introns, exons, intergenic, promoter regions, 3′- and 5′-UTRs; therefore they may represent antisense, bidirectional, or sense-overlapping sequences of specific genes [14, 15]. Several lncRNAs exhibit temporal and spatial expression patterns; moreover, their expression can be restricted to particular tissue or cell cycle stages, indicating diverse biological roles for lncRNAs [16]. Deregulation of distinct lncRNAs has been reported to promote tumor formation, progression, and metastasis in many types of cancer, including hematologic malignancies [17, 18]. The number of known human lncRNA transcripts is still evolving. LNCipedia v3.1 is the largest integrated repository containing 111,685 human annotated lncRNAs, with many loci generating multiple transcripts [19].

The knowledge of the role of lncRNAs in MM is virtually absent. A recent paper investigated lncRNAs as biomarkers for predicting survival in MM patients [20]; in addition, MALAT1 (metastasis-associated lung adenocarcinoma transcript 1), a putative oncogenic lncRNA overexpressed in several solid tumors [21, 22], has been found overexpressed in MM and may represent a marker to predict MM progression [23]. Moreover, it is expressed in bone marrow mononuclear cells of newly diagnosed myeloma patients [23]. Finally, preliminary evidence has suggested deregulated levels of circulating lncRNAs in MM patients compared to healthy donors [24].

The present study was aimed at investigating the lncRNA expression profiles in a large and representative cohort of PC dyscrasia, including MGUS, SMM, MM, and PCL patients, and in normal bone marrow PC controls. We have identified deregulated lncRNAs putatively associated with MM pathogenesis and possibly linked to the accepted multi-step process underlining the different stages of the disease.

## RESULTS

### LncRNA expression profiling in plasma cell dyscrasia

**Figure 1:**
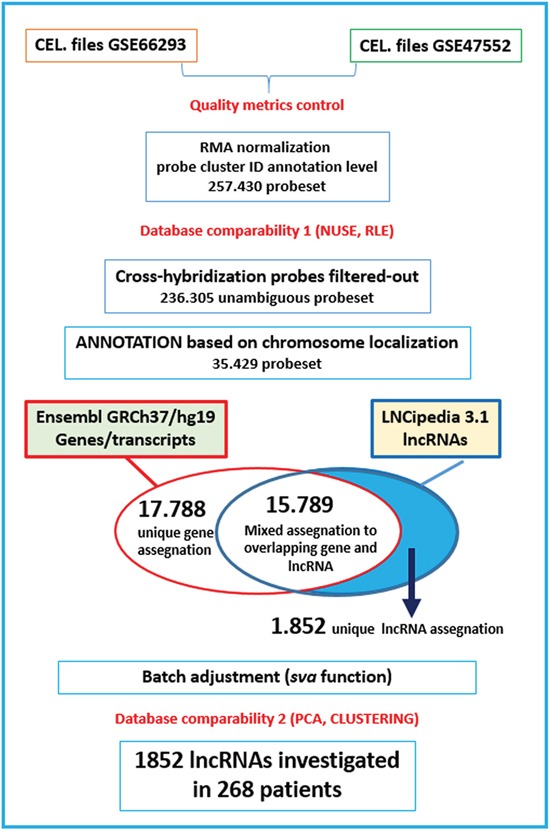
Custom annotation pipeline Flow diagram highlighting the different steps for processing the Gene 1.0 ST array data to investigate lncRNAs in combined databases (GSE66293 and GSE47552).

The expression profiles of lncRNAs have been investigated by Gene 1.0 ST array in a large cohort of 268 patients included in two different datasets and representative of the major forms of plasma cell dyscrasia. The panel totally included 20 MGUS, 33 SMM, 170 MM, 36 PCL patients and 9 normal bone marrow PCs samples. To be confident in detecting specifically lncRNAs, we applied a custom annotation pipeline able to remap the probes included in the original array to distinct lncRNAs according to the LNCipedia-v3.1 database (see Methods and Figure 1). Such a strategy allowed us to investigate the expression levels of 1852 well-annotated and specific human lncRNAs.

**Figure 2:**
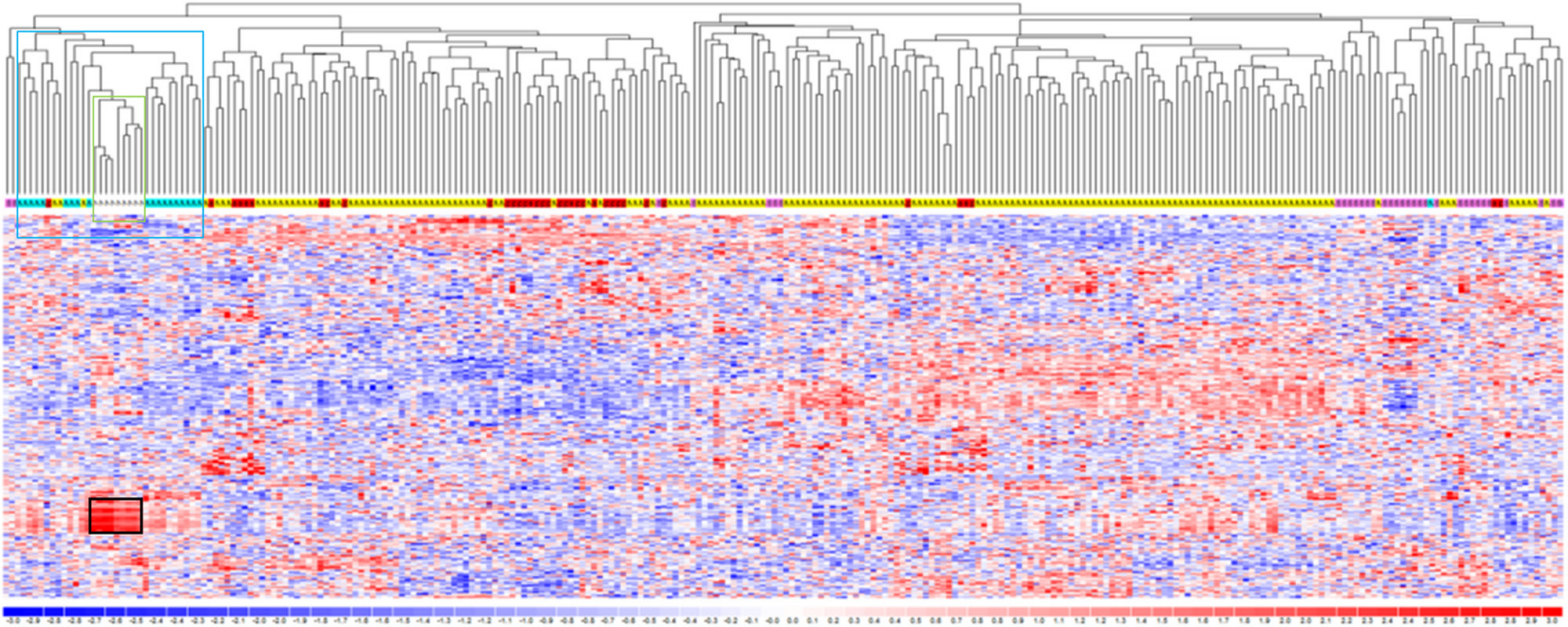
LncRNA expression profiling in plasma cell dyscrasia Hierarchical clustering of the 268 samples using the 230 most variable lncRNAs (patients in columns, lncRNAs in rows). The color scale bar represents the relative lncRNA expression changes normalized by the standard deviation. Color above the matrix indicates the type of samples: white, light blue, pink, yellow, and red represent Normal (N), MGUS, SMM, MM, and PCL samples, respectively. Specific types are enriched in colored sub-branches (see text). Black box identifies lncRNAs strongly upregulated in normal samples (see text).

To determine whether the natural grouping of lncRNAs expression profiling could be associated with a specific clinical entity, we performed an unsupervised analysis using conventional hierarchical agglomerative clustering of the 230 lncRNAs whose average change in expression levels varied at least 1.5-fold from the mean across the dataset (Figure 2). Notably, we found that all normal controls clustered together (cluster in the green box, *P* <0.0001), as did 19 of 20 MGUS cases (cluster in the blue box, *P* = 0.0011) and SMM patients (*P* = 0.0049). Notably, the lncRNAs strongly upregulated in normal samples (Figure 2, black box) are located in chromosomal regions, such as 14q32, 2p, and 22p, coding for the highly variable portions of the immunoglobulin genes or for IGV pseudogenes. We related this to the highly complex transcriptional activity of these regions in normal PCs to achieve polyclonal antibody repertoire, therefore excluded these transcripts from further analyses.

Next, we compared normal samples with MGUS, SMM, MM or PCL patients, and identified a set of 160 lncRNAs showing highly significant differential expression between normal PCs and those from the four different clinical entities (Supplementary Table S1). In particular, among this lncRNA panel, six transcripts were common to all the comparisons, whereas 25 lncRNAs resulted from at least three analyses (Table 1). Among these 31 lncRNAs, 19 were upregulated in the pathological samples compared to normal plasma cells; notably lnc-SCYL1-1, also known as *MALAT1*. This group also includes lnc-MON2-2, lnc-PTPDC1-7, and lnc-USP25-2, all of which overlapping host genes for members of the let-7 miRNA family, and lnc-USP25-2, lnc- APC6, lnc-LIPG-3, lnc-TRPV2-1 and lnc-C3orf25-2 that overlap host genes for small nucleolar RNAs (snoRNAs) molecules. Among the 19 upregulated lncRNAs, we found seven transcripts classified in Ensembl as pseudogenes; for each lncRNA/pseudogene, we looked for the corresponding putative parental gene when identifiable by the blat search (UCSC Blat tool at http://genome.ucsc.edu/index.html). Specifically, as shown in Table 1, lnc-C3orf25-2, lnc-MC2R-2, lnc-CHRDL2-3, lnc-SYT8-3, lnc-PAIP1-3, and lnc-ABCB5-3, are all pseudogenes of ribosomal protein encoding genes, whereas lnc-SAFB2-3 is a pseudogene of the *SNRPE* gene, which encodes a protein belonging to the large family of small nuclear ribonucleoproteins (snRNPs), the building blocks of the spliceosome complex. In addition, we found a significant upregulation of lnc-ANGPTL1-3 and lnc-RLIM-6 that map antisense to the Ras-specific guanine nucleotide-releasing factor *RALGPS2* and to the thyroid hormone transporter *SLC16A2*, respectively; and lnc-ARFIP1-5 localizing antisense to the E3 ubiquitin ligase *FBXW7* gene but also overlapping the *MIR4453* locus. Finally, lnc-SENP5-4 and lnc–AP1M2-1 localize head to head to the nuclear cap binding protein *NCBP2* gene, and interleukin enhancer binding factor 3 (*ILF3*), respectively. Among the 12 lncRNAs downregulated in the pathological samples compared to normal plasma cells, we found lnc-SNURF-1 and lnc-SNURF-3 that are host genes for SNORD115 and SNORD116 families. Two pseudogenes of the *RN7SL* gene at 14q21 were found also downregulated. This gene encodes the small cytoplasmic RNA component of the signal recognition particle (SRP) that associates with the ribosome and targets nascent proteins to the endoplasmic reticulum for secretion or membrane insertion. Other four pseudogenes are included in the downregulated group: lnc-STOM-7 that is a remnant of the Alpha-1,3-galactosyltransferase (*GGTA1*) gene encoding an enzyme present in most mammals except man; lnc-HSFY2-10 that is positioned in sense orientation to its parental *CD24* gene; lnc-CISH-3, and lnc-SEL1L3-6. Furthermore, we found the down-regulation of lnc-LRRC47-1, described below.

**Table 1:** Differentially expressed LncRNAs resulting from SAM analyses comparing N with MGUS, sMM, MM, or PCL patients

lncRNA	N	MGUS	SMM	MM	PCL	Chr	ALIAS; OVERLAPPING TRANSCRIPT^a^	Corr. lncRNA- transcript^b^	PsG^c^	Putative Parental Gene	Corr. lncRNA- parental gene^b^
**AC100793.1-1**	↑	↓	↓	↓	↓	17q21	RAMP2-AS1				
**ATL3-1**	↑	↓	↓	↓	↓	11q13	C11orf95				
**ADAP2-2**	↑	↓	↓	↓	↓	17q11	RN7SL138P		M	RN7SL (14q21)	NA
**STOM-7**	↑		↓	↓	↓	9q33	GGTA1P		UP		
**HSFY2-10**	↑		↓	↓	↓	Yq11	CD24P4; S to CD24	NA	PP	CD24 (Yq11)	NA
**CISH-3**	↑	↓		↓	↓	3p21	ZNF652P1; AS to DOCK3	R=0.053	PP	ZNF652 (17q21)	R=0.65 p<2.2e-16
**LRRC47-1**	↑	↓		↓	↓	1p36	TP73-AS1; AS to TP73	R=0.03			
**SEL1L3-6**	↑	↓	↓		↓	4p15			UnP	NA	
**SEMA4B-4**	↑	↓	↓		↓	15q26					
**PQLC2-5**	↑	↓	↓		↓	1p36	RN7SL277P; AS to CAPZB	R= − 0.26	M	RN7SL (14q21)	NA
**SNURF-1**	↑	↓	↓		↓	15q11	S to SNORD115-116 family	NA			
**SNURF-3**	↑	↓	↓		↓	15q11	S to SNORD115-116 family	NA			
**APC-6**	↓	↑	↑	↑	↑	5q22	EPB41L4A-AS1; S to SNORA13	NA			
**MON2-2**	↓	↑	↑	↑	↑	12q14	S to Let7i	NA			
**ARFIP1-5**	↓	↑	↑	↑	↑	4q13	AS to FBXW7, S to MIR4453	R=0.03 (FBXW7)			
**PTPDC1-7**	↓		↑	↑	↑	9q22	AS to MIRLET7DHG	NA			
**MC2R-2**	↓		↑	↑	↑	18p11			PP	RPL36A (Xq22)	NA
**SAFB2-3**	↓		↑	↑	↑	19p13	SNRPEP4		PP	SNRPE (1q32)	R=0.5 p =0.030
**SYT8-3**	↓		↑	↑	↑	11p15	RPL36AP39		PP	RPL36A (Xq22)	NA
**CHRDL2-3**	↓		↑	↑	↑	11q13			PP	RPS12 (6q23)	R=0.17
**SCYL1-1**	↓		↑	↑	↑	11q13	MALAT1				
**TRPV2-1**	↓		↑	↑	↑	17p11	LRRC75A-AS1; S to SNORD49B, 49A, 65	NA			
**LIPG-3**	↓		↑	↑	↑	18q21	SNHG22				
**USP25-2**	↓		↑	↑	↑	21q21	S to SNORD74, MIR99AHG	NA			
**C3orf25-2**	↓		↑	↑	↑	3q21	RPL32P3; S to SNORA7B	NA	UnP	RPL32 (3p25)	R=0.47 p<2.2e-16
**AP1M2-1**	↓		↑	↑	↑	19p13	ILF3-AS1				
**ANGPTL1-3**	↓		↑	↑	↑	1q25	AS to RALGPS2	R=0.3 p=3.8e-08			
**SENP5-4**	↓		↑	↑	↑	3q29	NCBP2 -AS2				
**PAIP1-3**	↓		↑	↑	↑	5p12	RPL29P12; AS to NNT	R= − 0.054	PP	RPL29 (3p21)	R=0.7 p<2.2e-16
**ABCB5-3**	↓		↑	↑	↑	7p21	RPL23P8		PP	RPL23 (17q12)	NA
**RLIM-6**	↓		↑	↑	↑	Xq13	AS to SLC16A2	R=0.37 p=3.7e-10			

Arrows indicate lncRNA expression, upregulation (↑) or downregulation (↓), in the corresponding group. Chromosomal localization (Chr), alias name, and overlapping transcripts are indicated.

a Sense (S) to, or Antisense (AS) to overlapping transcripts;

b Pearson correlation coefficient; NA =not available (not detected by the array);

c PsG=Pseudogene; Ensembl type: PP= Processed Pseudogene; UP= Unitary Pseudogene;UnP= Unprocessed Pseudogene; M= miscellaneous RNA.

### LncRNA expression in the different stages of the disease

In order to identify deregulated lncRNAs possibly associated with the different clinico-biological forms of the disease, we searched for lncRNAs whose expression was significantly and progressively increased/decreased from normal PCs to PCLs (Jonckheere–Terpstra test). As reported in Table 2 and Supplementary Figure S1, the expression levels of 15 lncRNAs progressively increased from normal to MGUS, SMM, MM and PCL samples. On the other hand, six lncRNAs displayed a significantly decreasing trend. From a structural point of view, the resulting 21 lncRNAs could be divided in two main categories: i.e. (i) lncRNAs that are transcribed as complex, interlaced networks of overlapping transcripts often including protein-coding genes (as specified in Table 2, fourth column); and (ii) those located and transcribed within the intergenic stretches of the genome.

**Table 2:** LncRNAs significantly increased/decreased in the progressive forms of plasma cell dyscrasias

lncRNAs SENSE or ANTISENSE (S or AS) TO ANNOTATED TRANSCRIPTS							
TREND	lncRNAs q<0.05^a^	Chr	ALIAS; OVERLAPPING TRANSCRIPTS^b^	Corr. lncRNA-overlapping transcript^c^	PsG^d^	Putative Parental Gene	Corr. lncRNA- parental gene^c^
↑	**RLIM-6**	Xq13	AS to SLC16A2	R=0.37 p=3.7e-10			
↑	**ANGPTL1-3**	1q25	AS to RALGPS2	R=0.3 p=3.8e-08			
↑	**SENP5-4**	3q29	NCBP2-AS2; S to NCBP2	R= − 0.054			
↓	**LRRC47-1**	1p36	TP73-AS1; AS to TP73	R=0.03			
↑	WHAMM-2	15q25	SNHG21; AS to FSD2	R= − 0.03			
↑	SERPINC1-1	1q25	GAS5; AS to ZBTB37	R= 0.29 p=1.1e-06			
↓	SNX29P2-3	16p11	AS to NPIPL1	NA	P	NA	
↓	**HSFY2-10**	Yq11	CD24P4; S to CD24	NA	PP	CD24 (Yq11)	NA
↑	CPSF2-2	14q32	AS to TRIP11	R= 0.063	PP	PTMA (2q37)	R= − 0.32 p=7.8e-08
**LINC**							
**TREND**	**lncRNAs q<0.05 ^a^**	**Chr**	**ALIAS**		**PsG^d^**	**Putative Parental Gene**	**Corr. lncRNA- parental gene^c^**
↓	IRF2-3	4q35					
↓	VKORC1L1-3	7q11	RP13-254B10.1		PP	NA	
↓	**STOM-7**	9q33	GGTA1P		UP	NA	
↑	RALGAPB-1	20q11	SNHG11				
↑	**MC2R-2**	18p11	RP11-681N23.1		PP	RPL36A (Xq22)	NA
↑	**SAFB2-3**	19p13	SNRPEP4		PP	SNRPE (1q32)	R=0.5 p=0.030
↑	KIF20B-7	10q23	SNRPD2P1		PP	SNRPD2 (19q13)	R=0.79 p<2.2e-16
↑	DNAJC16-1	1p36	CHCHD2P6 (447bp, and 462bp)		PP	CHCHD2 (7p11)	NA
↑	WDR11-7	10q26	RN7SKP167 RNA		M	RN7SK (6p12)	NA
↑	PNRC1-1	6q15	RN7SL336P		M	RN7SL (14q21)	NA
↑	DLX5-4	7q21	RN7SL252P		M	RN7SL (14q21)	NA
↑	ZC3H12B-10	Xq11	RN7SL799P		M	RN7SL (14q21)	NA

Arrows indicate lncRNA expression (upregulation ↑/downregulation ↓), from N to PCL samples. Chromosomal localization (Chr), alias name, and overlapping transcripts are indicated.

a lncRNAs also found in Table 1 are in bold;

b Sense (S) to, or Antisense (AS) to overlapping transcripts;

c Pearson correlation coefficient; NA=not available (not detected by the array);

d PsG=Pseudogene; Ensembl type: PP= Processed Pseudogene; UP= Unitary Pseudogene; UnP= Unprocessed Pseudogene; M= miscellaneous RNA.

Representative of the first group was lnc-LRRC47-1, whose expression showed a significant decreasing trend being significantly downregulated in MGUS, MM and PCL samples as compared to normal control (see above). In details, lnc-LRRC47-1 could be transcribed in 23 different transcripts, seven of which correspond to the reported TP73-AS1 transcripts that map antisense to the 3′UTR of the tumor suppressor *TP73* gene. In agreement with the previous analyses, we found a significant increasing expression trend of lnc-ANGPTL1-3, lnc-RLIM-6, and lnc-SENP5-4 from normal to pathological samples. Among the first group, we have also identified lnc-WHAMM-2 and lnc-SERPINC1-1, also known as SNHG21 and GAS5, respectively, which are host genes for SNORD molecules, and both showing a significant positive expression trend. Finally, this group also included three pseudogenes, the above described lnc-HSFY2-10, lnc-SNX29P2-3 that maps antisense to the *NPIPL1* gene encoding a nuclear pore complex-interacting protein member, and lnc-CPSF2-2, which is a pseudogene of Prothymosin alpha (*PTMA*) and is localized antisense to the thyroid hormone receptor interactor 11 (*TRIP11*) gene.

The second lncRNA group included 12 transcripts 10 of which are pseudogenes. Among the nine with a significant increasing expression trend from normal PCs to PCLs, we found lnc-PNRC1-1, lnc-DLX5-4, and lnc-ZC3H12B-10, which are all pseudogenes of the *RN7SL* gene. Lnc-SAFB2-3 and lnc-KIF20B-7 are pseudogenes of *SNRPE* and *SNRPD2*, respectively, which encode proteins included in the snRNPs; notably, the expression levels of these lncRNAs resulted significantly correlated with those of corresponding parental genes (Table 2, last column). Lnc-MC2R-2 and lnc-DNAJC16-1 are respectively processed pseudogenes of RPL36A gene and the transcription factor CHCHD2 that transactivates a conserved oxygen response element. Moreover, lnc-WDR11-7 is a pseudogene of the RN7SK family genes, involved in RNA Polymerase II activity control.

Finally, we investigated whether the lncRNAs that were gradually deregulated through the progressive stages of PC dyscrasia were also related to the disease progression in the same patient. To this regard, Gene Expression Profiling (GEP) data from a limited proprietary paired cohort of 19 MM patients at diagnosis and relapse/PCL progression corroborated the deregulated expression of lnc-SENP5-4, lnc-CPSF2-2, and lnc-LRRC47-1 during disease progression in this subset of patients (Supplementary Figure S2).

### LncRNA expression profiling in MM molecular subgroups

**Figure 3:**
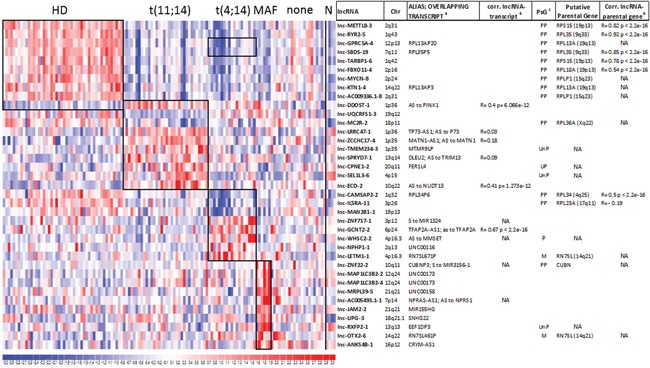
Identification of lncRNA signatures characterizing distinct MM genetic subgroups Heatmap of the differentially expressed lncRNAs in 129 MM patients stratified into the five molecular groups as specified in methods; black boxes indicate the ten most differentially expressed lncRNAs resulting from the supervised analysis comparing each subgroup *versus* the rest of the dataset. LncRNA expression levels in normal plasma cells are shown on the right. For each lncRNA, information about chromosomal localization (Chr), alias name, transcripts overlapping in sense or antisense (indicated as S or AS, respectively) direction, and the Pearson's correlation coefficient between lncRNA and the overlapping transcript (when detected by the array) are indicated. For lncRNAs/pseudogenes, the last column reports the putative parental gene and the Pearson's correlation coefficient between lncRNA and the parental gene expression when detected by the array. ^a^ Sense (S) to, or Antisense (AS) to overlapping transcripts; ^b^ NA indicates transcripts not detected by the array; ^c^ PsG =pseudogene; Ensembl type: PP= Processed Pseudogene; UP= Unitary Pseudogene; UnP= Unprocessed Pseudogene; M= miscellaneous RNA.

Based on the commonly accepted notion of the great heterogeneity in MM patients, we aimed at identifying lncRNAs deregulation distinctive of the major MM molecular subgroups. We focused on the proprietary panel of 129 MM patients, which is completely characterized for the major molecular alterations, i.e. chromosomal translocations involving the immunoglobulin heavy chain (IGH) locus, hyperdiploid (HD) status, deletions of 13q, 17p13, and gain of 1q. As shown in Figure 3 and Supplementary Table S2, among the 10 most differentially expressed lncRNAs in HD versus non HD (NHD) patients, we found nine upregulated lncRNAs that are all pseudogenes related to ribosomal protein coding genes; for these lncRNA-parental gene couples a highly positive correlation of their expression levels was found (Figure 3). Notably, in MM characterized by t(11;14) translocation the analysis unraveled the upregulation of lnc-LRRC47-1 and lnc-SEL1L3-6, and the downregulation of lnc-MC2R-2 and lnc-SPRYD7-2, the latter of which may have 25 putative transcripts, eight of which corresponding to *DLEU2*. Patients carrying t(4;14) translocation upregulated lnc-WHSC2-2, a pseudogene located in the intron 19 of *MMSET* gene, which encodes the histone methyltransferase deregulated as the result of t(4;14) translocation. Finally, MM samples with either the t(14;16) or t(14;20) translocations upregulated lnc-LIPG-3, the host gene of SCARNA17, and lnc-JAM2-2, which overlaps the *MIR155HG* locus.

Considering MM samples with 1q gain lesion, they specifically upregulated 7 lncRNAs all of which are located in the amplified chromosomal region and include lnc-SERPINC1-1 (*GAS5*) and lnc-CD46-4 that maps antisense to miR-102 gene. Patients with del13 significantly deregulated 14 lncRNAs, five of which reside on 13q14 region. It is worth mentioning the downregulation of lnc-SPRYD7-1, also known as *DLEU2*, whose expression significantly correlated with that of miR-15a and miR-16-1 located in the same region (Supplementary Figure S3). MM patients with del17 upregulated 3 lncRNAs (Table 3).

**Table 3:** LncRNAs significantly deregulated in MM patients with del13, del17 or 1q gain

lncRNA	Score(d)	Fold Change	Chr	ALIAS; OVERLAPPING TRANSCRIPT^a^	Corr. lncRNA-overlapping transcript^b^	PsG^c^	Putative Parental Gene	Corr. lncRNA- parental gene^b^
				**del17**				
**MEF2C-2**	4.98	2.26	5q14	LINC00461; S to MIR9-2	NA			
**TRPV4-1**	4.04	2.16	12q24	FAM222A-AS1; AS to FAM222A	R= − 0.07			
**ROPN1B-7**	3.62	1.60	3q21	FAM86JP		PP	FAM86C1 (11q13)	R= 0.22
				**1q gain**				
**TOR1AIP2-5**	3.81	1.48	1q25					
**ANGPTL1-3**	3.78	1.38	1q25	AS to RALGPS2	R=0.3 p=3.8e-08			
**CD46-4**	3.43	1.30	1q32	AS to miR-102	NA			
**SERPINC1-1**	3.24	1.38	1q25	GAS5; AS to ZBTB37	R= 0.29 p=1.1e-06			
**HNRNPU-1**	3.12	1.48	1q44	HNRNPU-AS1; AS to COX20	R= 0.18			
**TRAF5-1**	3.11	1.35	1q32	LINC00467				
**ANKRD36BP1-1**	2.90	1.30	1q24	ANKRD36BP1				
				**del13**				
**NEK3-1**	−4.07	0.70	13q14	MRPS31P5		UnP	NA	
**FAM60A-6**	−3.95	0.68	12p11	S to OVOS2	NA			
**FAM70B-1**	−3.67	0.79	13q14	GAS6-AS1; AS to GAS6	R= − 0.28			
**CYorf15A.1-2**	−3.30	0.39	Yq11	TXLNG2P		UnP	TXLNG (Xp22)	R= − 0.07
**MTRNR2L1-4**	−3.28	0.85	17p11			PP	MT-ND2	NA
**ZFYVE1-4**	−3.03	0.87	14q24	RN7SL586P		M	RN7SL (14q21)	NA
**RNASEH1-7**	−2.96	0.81	2p25	TMSB4XP2; AS to COLEC11	R= 0.19	UnP	TMSB4X (Xp22)	NA
**KDM5D-3**	−2.91	0.66	Yq11					
**SLC7A1-1**	−2.81	0.82	13q12	MTUS2-AS1; AS to MTUS2	R= 0.19			
**SPRYD7-1**	−2.70	0.72	13q14	DLEU2; AS to TRIM13	R= 0.09			
**RCOR3-1**	−2.67	0.87	1q32			PP	RPS25 (11q23)	NA
**BACH1-2**	−2.67	0.84	21q21	GRIK1-AS1, AS to GRIK1	R= 0.18			
**CLYBL-1**	−2.65	0.76	13q32	S to CLYBL	R= 0.24			
**POM121L2-2**	3.77	1.80	6p22	ZNF204P		PP	NA	

a Sense (S) to, or Antisense (AS) to overlapping transcripts

b Pearson correlation coefficient; NA =not available (not detected by the array);

c PsG=Pseudogene; Ensembl type: PP= Processed Pseudogene; UP= Unitary Pseudogene; UnP= Unprocessed Pseudogene; M= miscellaneous RNA.

### Correlation between lncRNA and gene expression levels

Based on expression levels analysis, we evaluated for each of the differentially expressed lncRNA reported in Tables 1-3: i) the correlation with annotated transcripts that mapped sense or antisense to it, i.e. a potential *in cis* regulation; and ii) the relationship between lncRNAs/pseudogenes and matching parental genes. The results, reported in Tables 1-3, pointed out that pseudogenes are often co-expressed at variable levels with their putative parental gene. Furthermore, to gain evidences of lncRNAs that may potentially act on gene expression, we extended this correlation analysis to all the 17788 transcripts unambiguously detectable by the arrays. To be confident of identifying lncRNA-gene real interaction, we focused on correlation coefficient > 0.9, and found five lncRNAs that highly correlated with one or more genes (Table 4), all of them modulated between normal and pathological samples. Among these, besides lnc-cYorf15A.1-2 and *KDM5D* gene that map ∼112 kb apart on chromosome Y, the other four lncRNAs correlated with genes located on different chromosomes, suggesting *in trans* interplay for such couples.

**Table 4:** Genes whose expression level highly correlate with that of lncRNAs resulting from SAM analyses

lncRNA	N	MGUS	SMM	MM	PCL	Chr	ALIAS	HIGHLY CORRELATED GENE	Corr. lncRNA- gene^a^
**C14orf49-4**	↑	↓				14q32	SNHG10	**RPL18A** (19p13)	0.907
**RYR2-5**	↓		↑	↑		1q43	RPL35P1	**RPL35** (9q33)	0.918
**TAMM41-3**	↑	↓				3p25	RN7SL147P	**OCEL1** (occludin/ELL domain containing 1; 19p13)	0.905
**CYorf15A.1-2**	↑				↓	Yq11	Taxilin gamma 2, pseudogene	**KDM5D** (LYSINE-SPECIFIC DEMETHYLASE 5D; Yq11)	0.943
								**USP9Y** (UBIQUITIN-SPECIFIC PROTEASE 9; Yq11)	0.910
**HSFY2-10**	↑		↓	↓	↓	Yq11	CD24P4	**BPI** (BACTERICIDAL PERMEABILITY-INCREASING PROTEIN; 20q11)	0.914
								**CEACAM8** (CARCINOEMBRYONIC ANTIGEN-RELATED CELL ADHESION MOLECULE 8; 19q13)	0.923

Arrows indicate lncRNA expression, upregulation (↑) or downregulation (↓), in the corresponding group. Chromosomal localization (Chr), alias name are indicated.

a Pearson correlation coefficient

### Quantitative RT-PCR validation of differentially expressed lncRNAs

**Figure 4:**
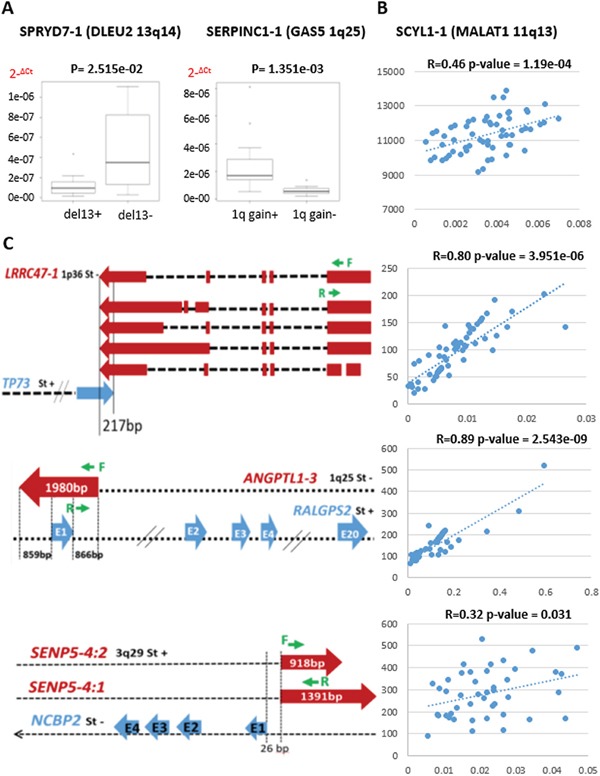
Quantitative RT-PCR validation of lncRNA expression in 60 MM cases **A.** Box plots of lnc-SPRYD7-1 and lnc-SERPINC1-1 expression show a significant correlation with group membership based on Wilcoxon rank-sum tests (*p*-values are shown above each panel). The expression levels are represented as 2^−ΔCt^. **B.** MALAT1 expression validation; Pearson's correlation coefficient was calculated between GEP data and quantitative RT-PCR results expressed as 2^−ΔCt^. **C.** Genomic map of lncRNAs (red) and antisense genes (blue) with custom primers (green) localization. Sense or antisense chromosomal position (St+ or St-, respectively) are specified. For lnc-LRRC47-1, the scheme was limited to longer transcripts. Pearson's correlation coefficient was calculated between GEP data and quantitative RT-PCR results expressed as 2^−ΔCt^.

The expression levels of six relevant lncRNAs found differentially expressed in our analyses were investigated by qRT-PCR performed in 60 MM samples for whom RNA material was available. Specifically, we validated the expression levels of the well-known MALAT1, GAS5, and DLEU2 lncRNAs. Furthermore PCR results confirmed the significant upregulation of GAS5 in samples with 1q gain lesion, and the downregulation of DLEU2 in patients carrying del13 (Figure 4A–4B). Likewise, we validated the expression of lnc-ANGPTL1-3, lnc-SENP5-4, and lnc-LRRC47-1, found progressively deregulated in association with more aggressive forms of the disease (Figure 4C).

### Functional annotation of MALAT1 signature in MM

**Figure 5:**
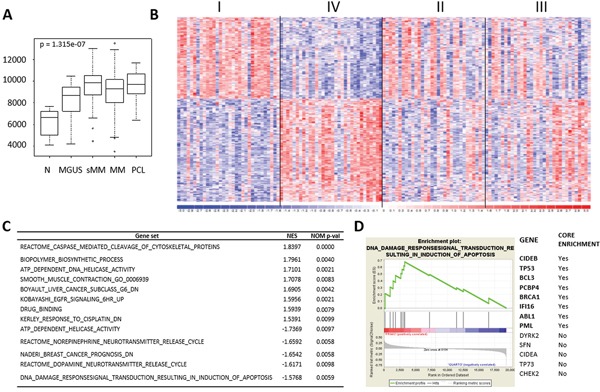
MALAT1 expression deregulation in MM patients **A.** Box plot of MALAT1 expression level in 9 N, 20 MGUS, 33 SMM, 170 MM, and 36 PCL samples, as detected by GEP; *p*-value was calculated by Kruskal-Wallis rank sum test. **B.** Heatmap of the 518 differentially expressed lncRNAs identified by comparing the first *vs* fourth quartile of 129 MM patients stratified into four groups based on *MALAT1* expression level. **C.** Gene sets significantly up- and down-regulated in MALAT1 quartile IV versus I. Gene sets were selected on nominal p-value<0.01 and are ordered according to NES value. **D.** Enrichment plot of “DNA damage response signal transduction resulting in induction of apoptosis” gene set detected by GSEA. The green curves show the enrichment score and reflect the degree to which each gene (black vertical lines) is represented at the bottom of the ranked gene list. Genes contributing to the core enrichment in the gene set are indicated in bold.

Although we found several lncRNAs deregulated in the different form of PC dyscrasia, only a very few number have been functionally validated in biological and disease processes. Among these, we found MALAT1, the abundant nuclear-retained lncRNA found overexpressed in several cancers associated with high proliferation and metastasis, although the underlying mechanism(s) behind this deregulation and its significance in tumorigenesis is still poorly understood. Our data demonstrates a significant overexpression of MALAT1 in tumor samples compared to healthy controls (Figure 5A). To gain insight into the role of MALAT1 in MM, we focused on the proprietary MM group (due to the absence of any bias related to sample molecular characteristics) and looked for the gene expression signature associated with MALAT1 expression. Specifically, we ranked patients in four classes based on MALAT1 expression level. Next, by comparing the less (I quartile) with the most (IV quartile) expressing patients, we found 518 genes differentially expressed between the two groups that appeared to be gradually modulated in the four quartiles (Figure 5B and Supplementary Table S3). Functional enrichment analysis of the 518 deregulated genes using ToppGene evidenced 88 significantly enriched pathways, many of which attributable to mRNA maturation, proteasome degradation, cell cycle processes, and p53-dependent G1/S DNA damage checkpoint (Supplementary Table S4). In addition, Gene Set Enrichment Analysis (GSEA) was used to identify a priori defined sets of genes showing concordant modulation between patients with low and high MALAT1 expression level. Notably, GSEA analysis identified 13 gene sets (Figure 5C and Supplementary Table S5) among which the pathway associated with p53-mediated DNA damage response was highly enriched in MM group expressing less MALAT1 (Figure 5D).

## DISCUSSION

LncRNAs are an emerging field of investigation as they are suggested to regulate key biological processes, including cellular proliferation and differentiation, and their aberrant expression being strongly associated with cancer [25].

To the best of our knowledge, this is the first report describing the global expression profiles of lncRNAs in a large cohort of samples representing all the major different forms of plasma cell dyscrasias. Our study identified 31 lncRNAs that were specifically deregulated in this pathology compared to normal bone marrow PC controls (Table 1). In particular, we found six lncRNAs deregulated in all the different clinical forms of the disease, whereas 18 others appear associated with more advanced phases of the disease, as their expression in MGUS samples does not differ significantly from normal controls. A very notable example among lncRNAs found upregulated in pathological samples, is *MALAT1* that has been widely reported to be upregulated in a large variety of solid tumors in association with progression and cancer metastasis [21]. It may be surprising that five lncRNAs are significantly downregulated in MGUS, SMM and PCL groups but not in MM one. This finding may be explained by the great heterogeneity of the MM patient. In agreement with this hypothesis, three out of the five lncRNAs, specifically lnc-SNURF-1, lnc-SNURF-3, and lnc-SEL1L3-6, were significantly deregulated in distinct MM subgroups (Supplementary Table S2).

Notably, we identified 21 lncRNAs whose expression was progressively deregulated in association with the increased aggressiveness of the disease (Table 2). Among these, the expression levels of lnc-SENP5-4, lnc-CPSF2-2, and lnc-LRRC47-1 were found to be significantly different in a cohort of 19 MM patients at diagnosis compared to the corresponding relapse/PCL progressed phases (Supplementary Figure S2), a finding that further supports the previous evidence. In addition, a recent study investigating lnc-LRRC47-1 in MM, confirmed its progressive downregulation from normal PCs to MGUS and symptomatic disease, which appeared independent from the methylation status of its promoter [26]. Thus, it is conceivable that these lncRNAs may have a role in the progression of the disease. Furthermore, disease progression is associated with the upregulation of *GAS5* and lnc-ANGPTL1-3, both located at 1q25; this finding is in line with the GEP-model for high-risk MM that is enriched in overexpressed genes mapping to chromosome 1q [27]. Certainly the role of *GAS5* in MM deserves further studies considering that almost all the existent literature describes *GAS5* down-regulation in different cancers, above all solid tumors; according to existing data, *GAS5* both inhibits the proliferation and promotes the apoptosis of multiple cell types, consistent with a tumor suppressor role (reviewed in [28]). To date, *GAS5* overexpression in tumor has been reported only in mesothelioma [29], where the Authors hypothesized, as a possible explanation of the discrepancy, that high *GAS5* expression in that cellular milieu does not only control cell cycle but also act as a natural sponge for miRNAs. *GAS5* has been demonstrated as host gene for snoRNA molecules that traditionally function as guide for the post-transcriptional modification of ribosomal and some spliceosomal RNAs and are supposed to play a role in alternative splicing processes [30]. According to this, we may hypothesize that in PC dyscrasia *GAS5* deregulation affects processes that represent oncogenic mechanisms in MM.

In the context of MM, we demonstrated that deregulated patterns of lncRNAs expression are associated with distinct molecular subtypes, as it has long been commonly accepted for mRNAs, miRNAs, and snoRNAs [30–33]. Despite the fact that the mechanism and the function of lncRNAs, as well as the consequences of their deregulation remain to be fully clarified, the transcriptional lncRNA signature of some MM subgroups may not be surprising. In fact, the most upregulated lncRNAs in HD tumors are all pseudogenes of parental ribosomal protein coding genes, with which they are co-expressed (Figure 3). These findings are in line with the global up-regulation of the translational machinery, including genes involved in protein biosynthesis, characterizing this molecular group [34]. Based on the suggestion that the presence of pseudogenes may serve to modulate the activity of their parental gene [35, 36], it is feasible that these lncRNAs/pseudogenes may act as indirect post-transcriptional regulators decoying ncRNA, in particular miRNAs that target the parental gene [36]. Very interesting is also the lnc-JAM2-2, found to be upregulated in MM patients with MAF deregulation; this lncRNA overlaps *MIR155HG* gene, i.e. the host gene of the miR-155 found also upregulated in translocated MAF samples [31]. Considering MM patients carrying t(4;14), the upregulation of lnc-WHSC2-2 that maps intronic and antisense to the translocation target gene *MMSET*, is relevant. Notably, the consistent deregulation of lnc-WHSC2-2 in translocated MM resembles that of the snoRNA ACA11 that maps in the adjacent 3′ intron of *MMSET* on the sense strand [30, 37]. It is conceivable that, as reported for ACA11 [37], lnc-WHSC2-2 may be a critical target of the t(4;14) translocation in MM with a specific oncogenic role. Finally, the 1q-gain MM group showed significantly upregulated seven lncRNAs all located at 1q region, suggesting that gene dose effect may also be a mechanism behind lncRNAs deregulation.

Although every resultant lncRNA from our study warrants further investigation to elucidate its possible biological role, some of them are suggestive of a pathological function merely by relying on their genomic position. In particular, the overexpression of lnc-PTPDC1-7, lnc-USP25-2, and lnc-MON2-2 that overlap host genes for members of the let-7 family may somehow be related to this miRNA downregulation often detected in cancer [38]. Other lncRNAs lie in proximity of genes already known in cancer and thus potentially involved in their regulation, for example lnc-LRRC47-1, which may regulate the tumor suppressor *TP73* gene [39].

At present, there is strong evidence of a major role for lncRNAs in tumorigenesis based on several hundred lncRNAs already identified as being aberrantly expressed in cancer [25]. However, only a few of these lncRNAs have been functionally validated in normal and pathological biologic processes. Among these, MALAT1 was reported to regulate cellular proliferation by modulating the expression and/or pre-mRNA processing of cell cycle-regulated transcription factors; moreover, transient overexpression of MALAT1 enhanced cellular proliferation in cell lines and tumor formation in nude mice, while its depletion in tumor cells reduced tumorigenicity [40, 41]. In line with these findings, our data evidenced a significant overexpression of MALAT1 in tumor samples compared to healthy controls. Interestingly, functional annotation of the MALAT1 signature in MM identified enriched pathways linked to mRNA maturation, proteasome degradation, cell cycle processes, and p53-dependent G1/S DNA damage checkpoint. In addition, GSEA results showed that the pathway associated with p53-mediated DNA damage response was highly enriched in MM group less expressing MALAT1. This evidence suggests that p53 may be an important effector of MALAT1 function in PCs as it has been described in fibroblast [42].

In conclusion, our study reported many lncRNAs deregulated in the different forms of plasma cell dyscrasia. The challenge now and in the future is to identify the truly oncogenic and functionally relevant lncRNAs.

## MATERIALS AND METHODS

### Samples

The study was performed in a cohort of 268 patients included in two different datasets, and representative of all the major forms of plasma cell dyscrasia. Specifically, the proprietary dataset publicly available at the NCBI Gene Expression Omnibus repository (accession #GSE66293) included 4 normal controls (Voden, Medical Instruments IT), 129 MM, 24 pPCL, and 12 sPCL patients; the other [43] included 5 normal controls, 20 MGUS, 33 SMM, and 41 MM patients, whose expression data were publicly available (accession #GSE47552). With the exception of sPCL, the cohort consists of newly-diagnosed patients. PCs were purified from bone marrow samples using CD138 immunomagnetic microbeads (MidiMACS system, Miltenyi Biotec, Auburn, CA) and the purity of the positively selected PCs was >90% in all cases.

The proprietary 129 MM tumors employed for the study were representative of the major molecular characteristics of the disease. Samples were characterized for the presence of the most frequent chromosomal translocations and the ploidy status based on fluorescence in situ hybridization (FISH) evaluation criteria, as previously described [34]. Specifically, forty-eight showed a HD status; thirty-four samples were characterized by the t(11;14) or t(6;14) translocations; nineteen had the t(4;14) translocation; six had either the t(14;16) or t(14;20) translocations; and twenty-two did not fall into any of the other groups. Deletions of 17p13, 13q14 and gain of 1q were also evaluated by FISH.

### Expression profiling

GEP of the 268 samples was generated using GeneChip® Gene 1.0 ST Array (Affymetrix Inc., Santa Clara, CA) as previously described [33] (Supplementary Methods). Then, we applied a custom pipeline (Figure 1) to detect specific lncRNAs. Log2-transformed expression values were extracted from CEL files and normalized using RMA procedure in Expression Console Software (Affymetrix, Inc.) at the probe cluster ID annotation level. Cross-hybridization probes were filtered-out. To avoid potential biases due to the mixing of two different datasets, a quality control of the array datasets was performed. In particular, before proceeding, to prevent the inclusion of low-quality or non-reproducible data, normalized unscaled standard error (NUSE) and relative log-expression (RLE) distributions were generated in *aroma.affymetrix* package for all samples (samples would be removed if the 25th or the 75th percentile of NUSE and RLE exceeded the value of ±1.05 or ±0.5, respectively; Supplementary Figure S4A-B). Then, we combined annotated probes with both Ensembl transcripts (GRCh37/hg19 assembly) and lncRNAs from LNCipedia repository (http://www.lncipedia.org/), based on the chromosome localization of the target sequence identified by each probe; afterwards, we considered only probes univocally referable to lncRNA transcripts (i.e. that do not overlap with Ensembl transcripts). To summarize probes related to each lncRNA, we considered their median expression value. Finally, we added a further step of batch adjustment using the *sva* function in the homonymous package of the R software, to avoid any possible cohort-specific bias due to the merging of different datasets. Through this annotation pipeline, we selectively detected 1852 lncRNAs from LNCipedia database.

The principal component analysis (PCA) of the samples was performed by singular value decomposition of the considered data expression matrix using the *prcomp* function in the *stats* package, and the results were visualized using the *plot3d* function in the *rgl* package of the R software (Supplementary Figure S4C). Supervised analyses were carried out using the Significant Analysis of Microarrays software version 5.00 [44] using the web application provided in the *shiny* package of the R software (https://github.com/MikeJSeo/SAM). The cutoff point for statistical significance (at a *q*-value 0) was determined by tuning the Δ parameter on the false discovery rate and controlling the q-value of the selected probes. Hierarchical agglomerative clustering of patients based on the most significant probesets found was performed adopting Pearson's correlation and average as distance and linkage methods, respectively. DNA-Chip Analyzer software (dChip) [45] was used to perform clustering and graphically represent it. The list of differentially expressed genes was submitted to the ToppGene Suite portal (http://toppgene.cchmc.org) for functional enrichment analysis using the ToppFun application [46]. Microarray data were globally analyzed by Gene Set Enrichment Analysis (GSEA) [47]. MiRNA expression data was available for 125 samples out of our series (GSE70254 and GSE73452) generated using GeneChip® miRNA 3.0 Array (Affymetrix Inc., Santa Clara, CA) as previously described [48].

### Quantitative real-rime PCR (qRT-PCR)

For qRT-PCR, 100 ng of total RNA underwent reverse transcription using random primer mix and reagents from INVITROGEN (Life Technologies, Foster City, CA). Real-time PCR was performed in triplicate using TaqMan® Non-coding RNA Assays (Hs00273907_s1, Hs03671981_s1, and Hs00863925_m1 for MALAT1, GAS5, and DLEU, respectively) together with the TaqMan® Fast Universal PCR Master Mix on an Applied Biosystems 7900 Sequence Detection System. RNA samples were normalized based on the *house-keeping 18S* rRNA. Real-time PCR to validate lnc-LRRC47-1, lnc-ANGPTL1-3, and lnc-SENP5-4 was performed using 10 ng of total RNA and SYBR™ Green master mixes with custom primers (Supplementary Table S6). RNA samples were normalized based on the *GAPDH* gene. The threshold cycle (C_T_) was defined as the fractional cycle number at which the fluorescence passes the fixed threshold. LncRNA expression was relatively quantified using the 2^−ΔCt^ method (Applied Biosystems User Bulletin No. 2), and expressed as the relative quantity of target lncRNA normalized to the *house-keeping 18S* rRNA.

### Statistical analysis

Conventional statistical procedures were applied using standard packages of the R software (Kendall τ correlations and Wilcoxon rank-sum tests). The Jonckheere–Terpstra test function was used in the *clinfun* package to investigate the significance of the trend from normal donors through PCL cases in the expression levels of the selected lncRNA list generated on Gene 1.0 ST array. For robustness, to reduce biases that might be due to numerical imbalances within the groups (with the MM group being largely overrepresented) and to gain the most significant trends, an additional criterion was imposed that only steadily ascending or descending median values were allowed. In addition, a differential expression should be observed in at least 1 condition (Kruskal–Wallis tests using the appropriate function in the *stat* package in R software). The Benjamini and Hochberg correction was used to adjust significance of multiple tests (adjusted *p* < 0.05).

## SUPPLEMENTARY FIGURES AND TABLES




