# Supplementary material for: Distinct lncRNA transcriptional fingerprints characterize progressive stages of multiple myeloma
Source: Oncotarget. 2016 Feb 17;7(12):14814–30. doi: 10.18632/oncotarget.7442 (PMC4924754; doi:10.18632/oncotarget.7442)
Supplement: Supplementary file 1 [file oncotarget-07-14814-s001.pdf]

SUPPLEMENTARY FIGURES AND TABLES

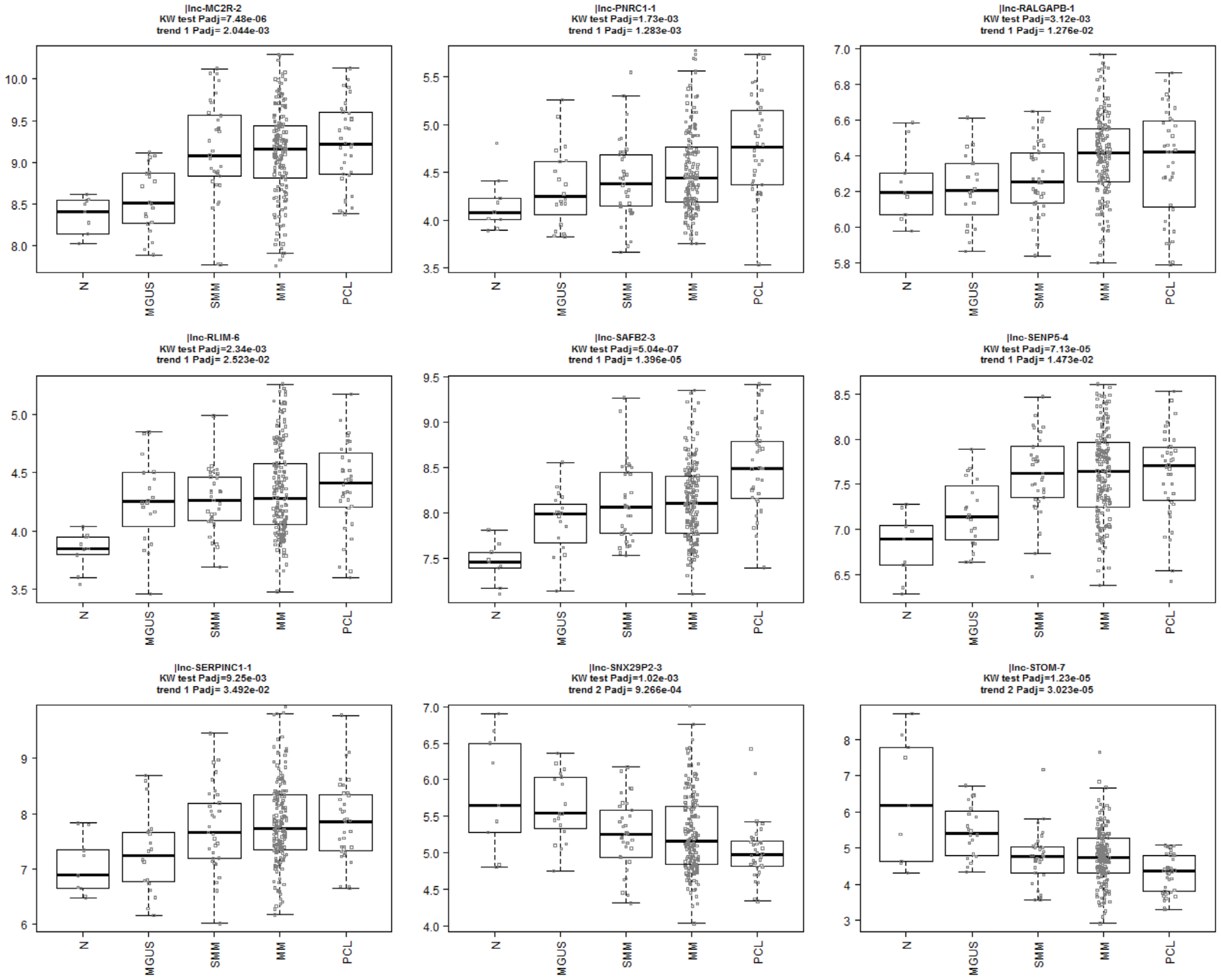

(continued)

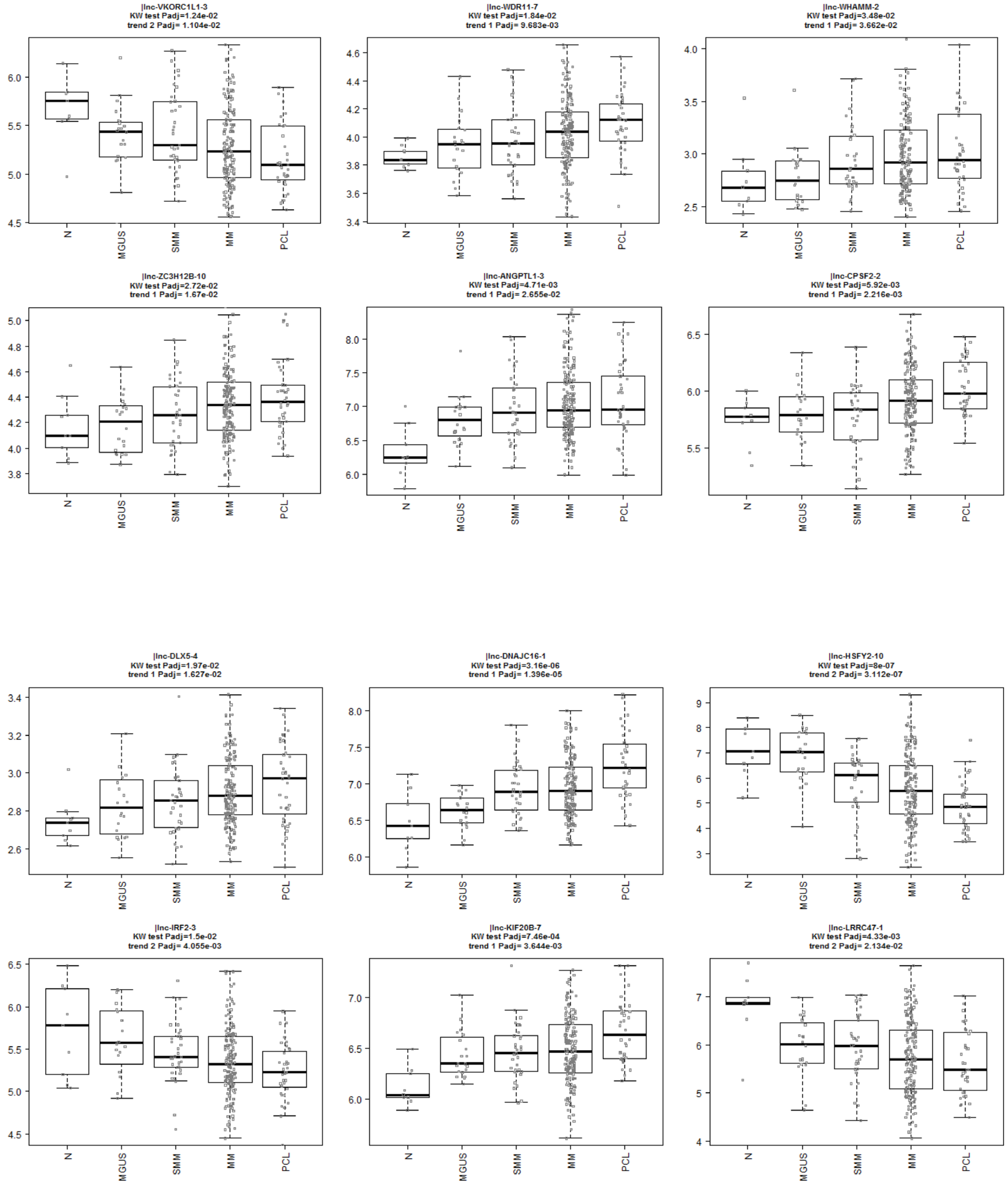

Supplementary Figure S1: Box plot of lncRNAs resulting from The Jonckheere–Terpstra test.

lncRNA	PAIRED t-TEST E *vs* REC *p*-value
lnc-ANGPTL1-3	0.3
**lnc-CPSF2-2**	**0.027**
lnc-DLX5-4	0.89
lnc-DNAJC16-1	0.94
lnc-HSFY2-10	0.8
lnc-IRF2-3	0.58
lnc-KIF20B-7	0.85
**lnc-LRRC47-1**	**0.007**
lnc-MC2R-2	0.98
lnc-PNRC1-1	0.87
lnc-RALGAPB-1	0.12
lnc-RLIM-6	0.97
lnc-SAFB2-3	0.92
**lnc-SENP5-4**	**0.04**
lnc-SERPINC1-1	0.069
lnc-SNX29P2-3	0.69
lnc-STOM-7	0.66
lnc-VKORC1L1-3	0.98
lnc-WDR11-7	0.23
lnc-WHAMM-2	0.99
lnc-ZC3H12B-10	0.18

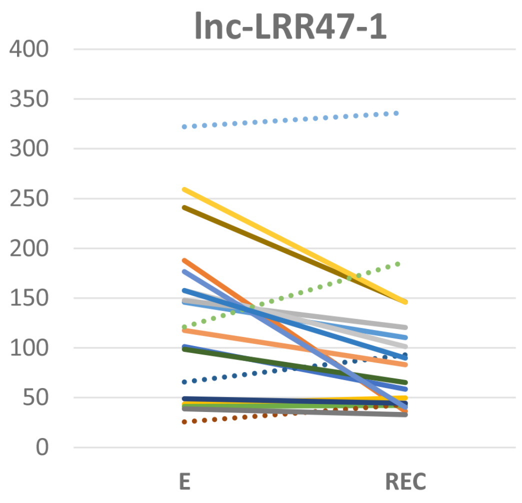

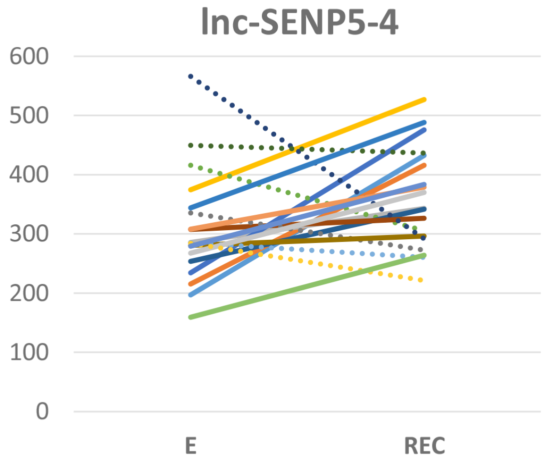

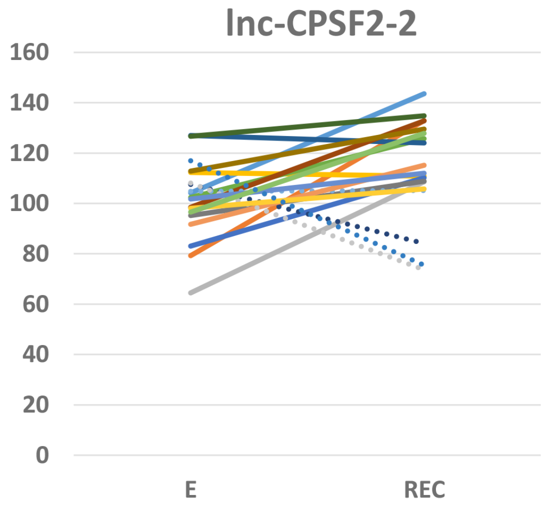

Supplementary Figure S2: Three out of 21 lncRNAs progressively increased/decreased in association with the stages of the desease are also significantly deregulated in a paired cohort of 19 MM and PCL.

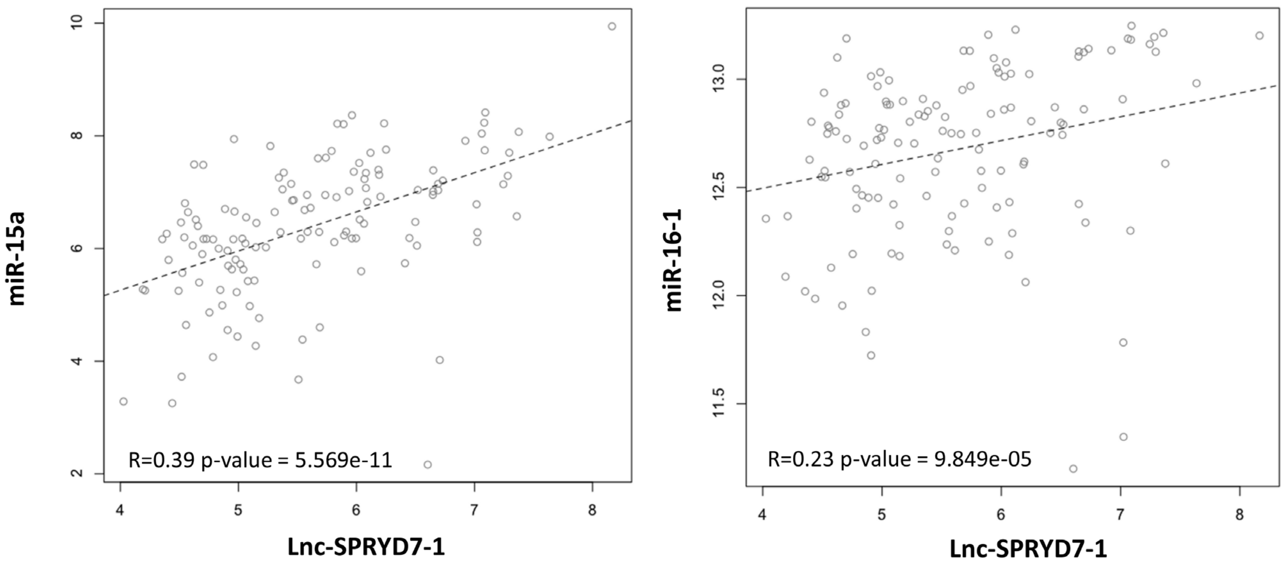

Supplementary Figure S3: Positive Pearson's correlation between lnc-SPRYD7-1 and miR-15a or miR-16-1 expression levels. MiRNA expression data was available for 125 samples out of our series (GSE70254 and GSE73452) generated using GeneChip® miRNA 3.0 Array (Affymetrix Inc., Santa Clara, CA) as previously described (Calura et al 2015)

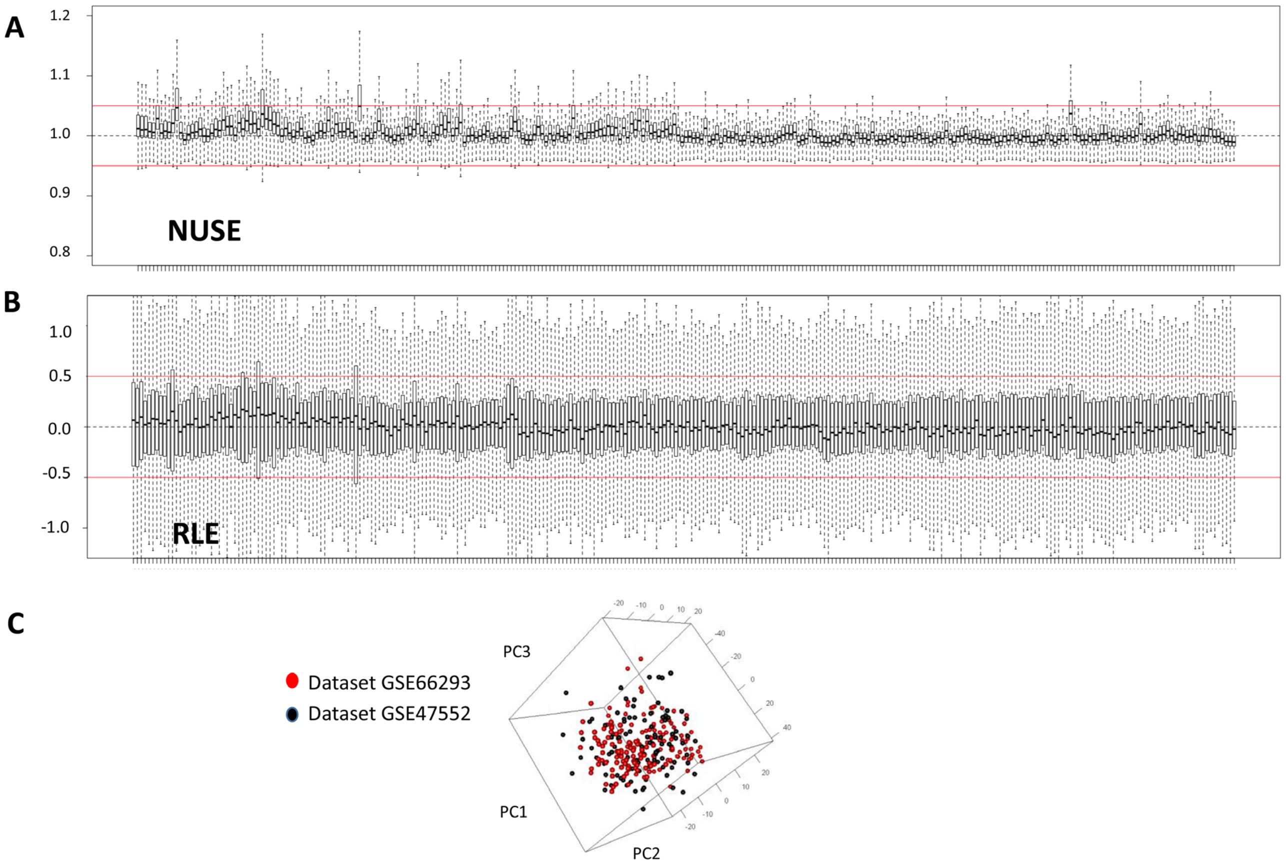

Supplementary Figure S4: Database quality control. **A-B.** Normalized unscaled standard error (NUSE) plot and relative log-expression (RLE) distribution plots. **C.** Principal Component analysis (PCA) plot.

Supplementary Table S1: Modulated lncRNAs between N controls and MGUS, sMM, MM or PCL patients.

See Supplementary File 1

Supplementary Table S2A: Modulated lncRNAs between HD+ and HD- MM patients.

See Supplementary File 1

Supplementary Table S2B: Modulated lncRNAs between t(11;14)+ and t(11;14)- MM patients.

See Supplementary File 1

Supplementary Table S2C: Modulated lncRNAs between t(4;14)+ and t(4;14)- MM patients.

See Supplementary File 1

Supplementary Table S2D: Modulated lncRNAs between MAF+ and MAF−MM patients.

See Supplementary File 1

Supplementary Table S3: List of 518 differentially expressed genes in MALAT quartile I vs quartile IV by SAM analysis at high stringency level (median FDR=0, 90th perc FDR=0). Genes are ordered according to SAM score, the fold change (FC) is also reported.

See Supplementary File 1

Supplementary Table S4: List of the significantly enriched pathways for the 518 differentially expressed genes, by Toppgene suite analysis.

See Supplementary File 1

Supplementary Table S5: Selected gene sets significantly up- and down-regulated in MALAT1 quartile IV versus I by GSEA analysis. Genes contributing to the core enrichment in each gene set are indicated in bold.

See Supplementary File 1

Supplementary Table S6: Sequence-specific SYBR green qRT-PCR primers.

See Supplementary File 1
