# Supplementary material for: Distinct lncRNA transcriptional fingerprints characterize progressive stages of multiple myeloma
Source: Oncotarget. 2016 Feb 17;7(12):14814–30. doi: 10.18632/oncotarget.7442 (PMC4924754; doi:10.18632/oncotarget.7442)
Supplement: Supplementary file 2 [file oncotarget-07-14814-s002.pdf]

Supplementary Table S1 : Modulated lncRNAs between N controls and MGUS, sMM, MM or PCL patients.

N vs MGUS

Gene Name	Score(d)	Fold Change
lnc-APC-6	4.33	2.76
lnc-MON2-2	4.12	1.68
lnc-ARFIP1-5	3.90	1.70
lnc-PTGDS-1	3.58	1.57
lnc-SNURF-1	-5.17	0.29
lnc-CISH-3	-4.90	0.60
lnc-SNURF-3	-4.57	0.30
lnc-DIRAS2-6	-4.50	0.48
lnc-ULBP3-3	-4.49	0.45
lnc-GALNT7-3	-4.44	0.39
lnc-SEMA4B-4	-4.36	0.62
lnc-KIAA1755-3	-4.34	0.40
lnc-NVL-6	-4.32	0.49
lnc-AC100793.1-1	-4.28	0.49
lnc-TMEM75-5	-4.17	0.44
lnc-ADAP2-2	-3.96	0.59
lnc-ANAPC7-1	-3.85	0.51
lnc-IL17RA-4	-3.81	0.49
lnc-LMTK2-3	-3.80	0.56
lnc-RNF135-1	-3.73	0.48
lnc-PLXNC1-4	-3.72	0.63
lnc-SEL1L3-6	-3.70	0.37
lnc-PQLC2-5	-3.69	0.54
lnc-B3GNT5-1	-3.69	0.61
lnc-XRN2-2	-3.66	0.65
lnc-C20orf173-4	-3.62	0.34
lnc-OR2AG2-1	-3.50	0.45
lnc-ZNF746-3	-3.45	0.60
lnc-C14orf49-4	-3.34	0.46
lnc-ZNF716-15	-3.34	0.44
lnc-SNURF-2	-3.21	0.37
lnc-PLA2G5-2	-3.20	0.71
lnc-KCNMA1-6	-3.19	0.73
lnc-TMEM14E-8	-3.18	0.58
lnc-CA14-3	-3.18	0.61
lnc-ENDOD1-7	-3.16	0.66
lnc-ATL3-1	-3.16	0.58
lnc-REST-8	-3.15	0.64
lnc-PKIB-1	-3.09	0.62
lnc-GGCX-2	-3.07	0.59
lnc-ZNF583-4	-3.04	0.54
lnc-AKR7A3-1	-3.01	0.59
lnc-SNHG15-1	-2.99	0.50
lnc-LHFPL3-2	-2.98	0.70
lnc-DDOST-1	-2.98	0.61
lnc-BCOR-8	-2.97	0.62
lnc-PUS10-3	-2.95	0.62
lnc-BTD-2	-2.94	0.65
lnc-TRIM74-4	-2.90	0.70
lnc-ELMOD2-2	-2.90	0.73
lnc-ANKRD9-4	-2.89	0.68
lnc-NSUN2-4	-2.89	0.78
lnc-CCNB1IP1-1	-2.89	0.61
lnc-LRRC47-1	-2.86	0.55
lnc-CPNE1-2	-2.81	0.46
lnc-POLDIP3-2	-2.78	0.63
lnc-HEXB-3	-2.78	0.70
lnc-TAMM41-3	-2.78	0.56
lnc-PGBD1-1	-2.77	0.66
lnc-DEPTOR-2	-2.74	0.79
lnc-FMNL1-1	-2.73	0.70

N vs sMM

Gene Name	Score(d)	Fold Change
lnc-APC-6	7.82	5.55
lnc-SCYL1-1	5.03	1.56
lnc-C3orf25-2	4.72	1.66
lnc-TRPV2-1	4.20	2.05
lnc-AP1M2-1	4.17	2.53
lnc-IL5RA-11	4.13	2.04
lnc-FDXACB1-2	4.13	1.67
lnc-METTL8-3	4.09	2.26
lnc-ABCB5-3	3.96	2.24
lnc-SENP5-4	3.93	1.78
lnc-HNRNPU-1	3.88	2.77
lnc-FOXM1-4	3.74	1.90
lnc-PACRGL-9	3.68	1.60
lnc-LIPG-3	3.60	2.79
lnc-F9-4	3.58	1.57
lnc-MC2R-2	3.57	1.87
lnc-SAFB2-3	3.55	1.60
lnc-CAMSAP2-2	3.53	1.58
lnc-C1orf132-1	3.53	2.65
lnc-MMADHC-8	3.44	1.43
lnc-PTPDC1-7	3.34	1.51
lnc-MON2-2	3.32	1.43
lnc-CHRDL2-3	3.27	1.90
lnc-RLIM-6	3.25	1.35
lnc-RYR2-5	3.18	1.78
lnc-AC009336.1-	3.17	1.53
lnc-BZRAP1-1	3.16	2.66
lnc-WDR74-1	3.14	1.96
lnc-TARBP1-6	3.13	1.54
lnc-GCNT4-4	3.13	2.18
lnc-UCP2-1	3.10	1.44
lnc-CRNKL1-4	3.06	1.58
lnc-USP25-2	3.05	1.86
lnc-PAIP1-3	3.01	1.53
lnc-PAN3-2	2.97	1.34
lnc-ANGPTL1-3	2.88	1.62
lnc-KIAA1755-5	2.85	1.74
lnc-SYT8-3	2.82	1.34
lnc-LETM1-1	2.80	1.34
lnc-ARFIP1-5	2.80	1.92
lnc-C12orf50-6	2.79	1.63
lnc-TOR1AIP2-5	2.77	1.85
lnc-ATL3-1	-7.86	0.43
lnc-ADAP2-2	-4.42	0.62
lnc-AC100793.1-	-4.38	0.49
lnc-LMTK2-3	-4.25	0.57
lnc-STOM-7	-3.89	0.33
lnc-PLXNC1-4	-3.85	0.67
lnc-SNURF-1	-3.70	0.42
lnc-PQLC2-5	-3.66	0.59
lnc-SNURF-3	-3.64	0.38
lnc-SEMA4B-4	-3.46	0.70
lnc-ANAPC7-1	-3.40	0.58
lnc-GGCX-2	-3.40	0.61
lnc-SEL1L3-6	-3.37	0.43
lnc-AGXT2L1-1	-3.25	0.67
lnc-HSFY2-10	-3.22	0.29
lnc-GALNT7-3	-3.17	0.55
lnc-LHFPL3-2	-3.10	0.71
lnc-IL17RA-4	-3.05	0.58

N vs MM

Gene Name	Score(d)	Fold Change
lnc-APC-6	6.30	4.25
lnc-TRPV2-1	4.17	2.11
lnc-TARBP1-6	4.17	1.74
lnc-MON2-2	3.85	1.58
lnc-AP1M2-1	3.80	2.06
lnc-RYR2-5	3.74	1.89
lnc-KIAA1755-5	3.73	2.02
lnc-CHRDL2-3	3.65	1.86
lnc-SENP5-4	3.62	1.72
lnc-AC090699.1.1-1	3.62	1.85
lnc-METTL8-3	3.55	1.96
lnc-FAM22E-2	3.55	2.35
lnc-ARFIP1-5	3.52	1.80
lnc-ABCB5-3	3.51	1.95
lnc-AC009336.1-8	3.51	1.52
lnc-CRNKL1-4	3.50	1.60
lnc-SAFB2-3	3.50	1.60
lnc-MC2R-2	3.49	1.81
lnc-SCYL1-1	3.49	1.38
lnc-NTNG1-2	3.39	2.20
lnc-UCP2-1	3.39	1.49
lnc-C3orf25-2	3.38	1.52
lnc-SBDS-19	3.38	1.93
lnc-TOR1AIP2-5	3.35	1.87
lnc-CAMSAP2-2	3.29	1.49
lnc-ANGPTL1-3	3.27	1.66
lnc-BARX2-3	3.27	1.43
lnc-LIPG-3	3.25	2.38
lnc-FOXM1-4	3.16	1.79
lnc-PTPDC1-7	3.15	1.45
lnc-AC078802.1-2	3.12	2.32
lnc-C1orf132-1	3.11	1.99
lnc-IL5RA-11	3.05	1.57
lnc-UXS1-6	3.05	1.32
lnc-HNRNPU-1	3.01	1.99
lnc-LETM1-1	3.01	1.41
lnc-PACRGL-9	3.00	1.49
lnc-POLR3G-1	2.98	1.87
lnc-RLIM-6	2.98	1.43
lnc-GPRC5A-4	2.97	1.58
lnc-BZRAP1-1	2.94	2.24
lnc-PAN3-2	2.92	1.37
lnc-OR5P3-1	2.87	1.73
lnc-MYCN-8	2.82	1.54
lnc-SRGAP3-1	2.80	1.56
lnc-GLRA2-2	2.79	1.41
lnc-DHX8-2	2.79	1.54
lnc-SYT8-3	2.75	1.37
lnc-PAIP1-3	2.64	1.45
lnc-DNAJC16-1	2.64	1.40
lnc-ZNF737-3	2.64	1.74
lnc-CXCR3-13	2.64	1.44
lnc-ROPN1B-7	2.60	1.38
lnc-SERPINC1-1	2.60	1.72
lnc-ESR2-1	2.58	1.52
lnc-C14orf126-4	2.58	1.41
lnc-RP11-168K9.2.1-6	2.55	1.25
lnc-TMPRSS2-4	2.55	1.43
lnc-ZSCAN2-5	2.55	1.46
lnc-MYO10-9	2.54	1.32
lnc-AIDA-1	2.52	1.78
lnc-TRIM28-1	2.52	1.38
lnc-BCL11A-7	2.49	1.37
lnc-TMEM111-1	2.49	1.43
lnc-SMTNL2-3	2.48	1.35
lnc-FDXACB1-2	2.48	1.36
lnc-USP25-2	2.47	1.84
lnc-RP11-110I1.12.1-3	2.46	1.28
lnc-SYNCRIP-2	2.46	1.63
lnc-CYorf17-1	2.45	1.58
lnc-AGBL1-4	2.45	2.14
lnc-GNAI2-1	2.45	1.36
lnc-ATL3-1	-5.15	0.51
lnc-ADAP2-2	-5.03	0.64
lnc-STOM-7	-4.88	0.33
lnc-AC100793.1-1	-4.32	0.53
lnc-CISH-3	-4.18	0.64
lnc-HSFY2-10	-3.77	0.26
lnc-LRRC47-1	-3.53	0.47
lnc-NVL-6	-3.47	0.57
lnc-TCL1B-2	-3.32	0.75

N vs PCL

Gene Name	Score(d)	Fold Change
lnc-APC-6	7.57	5.85
lnc-NTNG1-2	5.14	3.11
lnc-SAFB2-3	5.03	2.08
lnc-ABCB5-3	4.92	2.44
lnc-LIPG-3	4.86	4.07
lnc-PAN3-2	4.29	1.63
lnc-GBP2-1	4.17	3.17
lnc-MC2R-2	4.08	2.01
lnc-TRPV2-1	4.08	2.05
lnc-AP1M2-1	4.05	2.24
lnc-KBTBD2-2	3.94	1.88
lnc-DNAJC16-1	3.91	1.73
lnc-GCNT4-4	3.86	2.21
lnc-KIAA1755-5	3.86	2.33
lnc-SYNCRIP-2	3.86	2.09
lnc-MON2-2	3.75	1.90
lnc-PAIP1-3	3.73	1.63
lnc-SYT8-3	3.71	1.48
lnc-SENP5-4	3.71	1.74
lnc-KIF20B-7	3.66	1.46
lnc-BCL11A-7	3.58	1.68
lnc-TLE4-6	3.49	1.46
lnc-APPBP2-3	3.48	1.44
lnc-SCYL1-1	3.46	1.41
lnc-GGT1-5	3.43	1.59
lnc-C3orf25-2	3.37	1.52
lnc-CHRDL2-3	3.35	1.77
lnc-RLIM-6	3.32	1.47
lnc-USP25-2	3.31	2.21
lnc-VN1R2-4	3.31	1.61
lnc-SRGAP3-1	3.31	1.85
lnc-ZPLD1-7	3.26	1.28
lnc-TMEM111-1	3.24	1.56
lnc-OR5P3-1	3.22	1.89
lnc-PACRGL-9	3.22	1.47
lnc-OTUB2-1	3.17	1.38
lnc-ANGPTL1-3	3.16	1.69
lnc-UXS1-6	3.16	1.34
lnc-AC090699.1.1-1	3.11	1.99
lnc-CXCR3-13	3.10	1.67
lnc-PTPDC1-7	3.08	1.55
lnc-ZNF737-3	3.05	1.97
lnc-SMTNL2-3	2.99	1.54
lnc-EIF2B3-1	2.95	1.42
lnc-ARFIP1-5	2.95	1.87
lnc-CRNKL1-4	2.92	1.47
lnc-ATG12-3	2.89	1.52
lnc-GNAI2-1	2.87	1.43
lnc-KEAP1-1	2.84	1.79
lnc-PNRC1-1	2.81	1.51
lnc-MPDZ-8	2.80	1.32
lnc-ELOVL6-2	2.75	1.32
lnc-MYO10-9	2.74	1.35
lnc-CAMSAP2-2	2.70	1.36
lnc-ATL3-1	-5.86	0.48
lnc-SEL1L3-6	-5.85	0.17
lnc-HSFY2-10	-5.69	0.16
lnc-STOM-7	-5.58	0.24
lnc-SNURF-1	-5.21	0.27
lnc-SNURF-3	-5.10	0.25
lnc-AC100793.1-1	-4.82	0.50
lnc-ZNF716-15	-4.38	0.36
lnc-BACH1-1	-4.29	0.44
lnc-NVL-6	-4.24	0.47
lnc-ADAP2-2	-4.17	0.62
lnc-CISH-3	-4.05	0.61
lnc-LRRC47-1	-4.01	0.44
lnc-HEPH-1	-3.89	0.58
lnc-KDM5D-3	-3.64	0.47
lnc-OR2AG2-1	-3.54	0.37
lnc-GGCX-2	-3.47	0.56
lnc-SNX29P2-3	-3.47	0.58
lnc-TMEM75-5	-3.45	0.52
lnc-CYorf15A.1-2	-3.41	0.22
lnc-PGBD1-1	-3.39	0.58
lnc-PLXNC1-4	-3.22	0.66
lnc-PQLC2-5	-3.15	0.58
lnc-RNF135-1	-3.15	0.51
lnc-ANAPC7-1	-3.15	0.55
lnc-LHFPL3-2	-3.14	0.67
lnc-NBR2-1	-3.12	0.60
lnc-ULBP3-3	-3.08	0.53
lnc-DIRAS2-6	-3.06	0.57
lnc-SEMA4B-4	-3.02	0.67
lnc-LMTK2-3	-3.01	0.59

Supplementary Table S2A: Modulated lncRNAs between HD+ and HD- MM patients.

	Gene Name	Score(d)	Fold Change
HD +	lnc-METTL8-3	6.52	1.94
	lnc-RYR2-5	5.49	1.60
	lnc-SBDS-19	5.13	1.70
	lnc-TARBP1-6	4.99	1.43
	lnc-GPRC5A-4	4.92	1.51
	lnc-FBXO11-4	4.52	1.46
	lnc-MYCN-8	4.44	1.46
	lnc-KTN1-4	4.29	1.34
	lnc-AC009336.1-	4.27	1.32
	lnc-ALDH3B2-1	4.22	1.44
	lnc-PACRGL-11	4.20	3.28
	lnc-ZNF300-1	4.00	1.51
	lnc-IL5RA-11	3.94	1.37
	lnc-FAM65B-3	3.87	1.78
	lnc-CRNKL1-4	3.82	1.33
	lnc-CAMSAP2-2	3.63	1.28
	lnc-MC2R-2	3.57	1.43
	lnc-DHX8-2	3.55	1.34
	lnc-APC-6	3.41	1.56
	lnc-GUF1-7	3.35	1.28
	lnc-FAM110B-2	3.26	1.55
	lnc-PAIP1-3	3.18	1.26
	lnc-NKX2-4-5	3.09	1.35
	lnc-MUC15-3	3.04	1.40
	lnc-DAOA-10	2.94	1.20
	lnc-MAGEB5-6	2.93	1.30
	lnc-AL901608.1-	2.89	1.97
	lnc-SNURF-3	2.89	1.49
	lnc-DPF3-3	2.85	1.41
	lnc-ATG12-3	2.84	1.25
	lnc-TUSC3-3	2.81	1.17
	lnc-WBSCR17-1	2.80	1.33
	lnc-WDTC1-4	2.79	1.19
	lnc-SNURF-1	2.70	1.38
	lnc-NAPEPLD-5	2.69	1.46
	lnc-C9orf100-1	2.69	1.21
	lnc-MC5R-1	2.65	1.63
	lnc-BCL11A-7	2.64	1.22
	lnc-MAN2B1-1	2.62	1.28
	lnc-SMARCA5-4	2.62	1.21
	lnc-FDXACB1-2	2.58	1.22
	lnc-C3orf25-2	2.58	1.22
	lnc-TRPV2-1	2.56	1.29
	lnc-TRPV4-1	2.55	1.29
	lnc-DDOST-1	-4.53	0.65
	lnc-ZCCHC17-4	-3.73	0.73
	lnc-ECD-2	-3.65	0.72

Supplementary Table S2B: Modulated lncRNAs between t(11;14)+ and t(11;14)- MM patients.

	Gene Name	Score(d)	Fold Change
t(11;14) +	lnc-UQCRFS1-3	5.77	2.29
	lnc-MC2R-2	4.42	1.53
	lnc-ACTR1B-5	4.41	2.04
	lnc-C6orf183-1	4.39	1.34
	lnc-ZNF300-1	4.14	1.54
	lnc-RYR2-5	4.13	1.46
	lnc-METTL8-3	4.01	1.59
	lnc-BCL11A-7	3.96	1.33
	lnc-GUF1-7	3.94	1.34
	lnc-LINS-1	3.81	1.63
	lnc-MYCN-8	3.74	1.40
	lnc-DHX8-2	3.66	1.35
	lnc-AC009336.1-8	3.54	1.27
	lnc-WDR74-1	3.52	1.53
	lnc-TARBP1-6	3.44	1.31
	lnc-ABCB5-3	3.42	1.50
	lnc-NOL6-1	3.42	1.44
	lnc-SEMA3D-3	3.39	1.23
	lnc-EIF2B3-1	3.30	1.22
	lnc-MAGEB5-6	3.23	1.34
	lnc-SBDS-19	3.22	1.44
	lnc-AL901608.1-1	3.19	2.28
	lnc-PAIP1-3	3.18	1.27
	lnc-TRPV4-1	3.15	1.37
	lnc-CCNB1IP1-1	3.15	1.32
	lnc-ANKRD36BP1	3.08	1.35
	lnc-MC5R-1	3.05	1.79
	lnc-DUSP26-9	3.03	1.25
	lnc-FAM65B-3	3.02	1.62
	lnc-CHRDL2-3	3.02	1.36
	lnc-SYT8-3	3.00	1.21
	lnc-KTN1-4	3.00	1.25
	lnc-GPRC5A-4	2.94	1.30
	lnc-FAM110B-2	2.94	1.51
	lnc-TRPV2-1	2.94	1.34
	lnc-CRNKL1-4	2.89	1.25
	lnc-C11orf31-2	2.85	1.31
	lnc-MFSD4-8	2.81	1.27
	lnc-SMTNL2-3	2.79	1.20
	lnc-LGALS14-1	2.78	1.49
	lnc-WDR73-3	2.76	1.47
	lnc-FBXO11-4	2.72	1.28
	lnc-RBMXL3-2	2.70	1.18
	lnc-SDCBP-2	2.69	1.19
	lnc-POLR2J-1	2.68	1.22
	lnc-RP11-63E5.6.	2.65	1.19
	lnc-PRICKLE2-2	2.63	1.19
	lnc-LRRC47-1	-6.46	0.48
	lnc-ZCCHC17-4	-5.84	0.64
	lnc-DDOST-1	-5.36	0.60
	lnc-ECD-2	-5.25	0.63
	lnc-TMEM234-3	-5.09	0.63
	lnc-SPRYD7-1	-4.79	0.54
	lnc-CPNE1-2	-4.75	0.45
	lnc-SEL1L3-6	-4.39	0.33
	lnc-B4GALNT3-2	-4.36	0.68
	lnc-C20orf173-4	-4.36	0.48
	lnc-SERPINB9-1	-4.36	0.60
	lnc-MARVELD3-3	-4.36	0.59
	lnc-WRNIP1-36	-4.36	0.69
	lnc-SYT16-2	-4.36	0.71
	lnc-ACOT1-2	-4.36	0.81
	lnc-NMUR1-1	-4.36	0.67
	lnc-DEF8-2	-4.36	0.78
	lnc-UHRF1BP1L-2	-4.36	0.82
	lnc-BCL2L13-1	-4.36	0.73
	lnc-FAM60A-6	-4.36	0.71
	lnc-MYO10-1	-4.36	0.77
	lnc-CIAO1-1	-4.36	0.77

Supplementary Table S2C: Modulated lncRNAs between t(4;14)+ and t(4;14)- MM patients.

	Gene Name	Score(d)	Fold Change
t(4;14) +	lnc-CAMSAP2-2	4.59	1.46
	lnc-IL5RA-11	4.40	1.54
	lnc-MAN2B1-1	4.29	1.63
	lnc-SBDS-19	4.10	1.75
	lnc-GPRC5A-4	4.04	1.55
	lnc-GREB1-10	3.96	1.53
	lnc-NKX2-4-5	3.88	1.60
	lnc-ATG12-3	3.87	1.46
	lnc-RYR2-5	3.63	1.53
	lnc-FBXO11-4	3.60	1.49
	lnc-GPR160-1	3.57	1.68
	lnc-XRN2-2	3.42	1.36
	lnc-FDXACB1-2	3.39	1.38
	lnc-ZSCAN2-5	3.37	1.44
	lnc-KTN1-4	3.28	1.35
	lnc-C14orf49-4	3.23	1.99
	lnc-CRNKL1-4	3.07	1.34
	lnc-METTL8-3	3.01	1.56
	lnc-TBPL2-1	2.97	1.57
	lnc-WDTC1-4	2.93	1.25
	lnc-RP1-241P17.[illegible]	2.92	1.22
	lnc-RCOR3-1	2.89	1.23
	lnc-NFKB1-4	2.78	1.30
	lnc-IRG1-2	2.76	1.22
	lnc-DPF3-3	2.75	1.53
	lnc-IRF2-3	2.74	1.31
	lnc-ALDH3B2-1	2.73	1.38
	lnc-NBN-2	2.71	1.51
	lnc-PSMB7-1	2.70	1.38
	lnc-OR2AG2-1	2.67	1.81
	lnc-ZNF716-15	2.67	1.57
	lnc-LRRC47-1	2.63	1.54
	lnc-RPS4XP21-6	2.63	1.21
	lnc-ACO1-6	2.62	1.52
	lnc-YBEY-2	2.62	1.33
	lnc-CUX1-2	2.59	1.29
	lnc-NEK3-1	2.58	1.38
	lnc-GCNT2-2	-4.65	0.44
	lnc-ZNF717-1	-4.30	0.64
	lnc-WHSC2-2	-4.26	0.78
	lnc-LETM1-1	-4.18	0.70
	lnc-NPHP1-1	-3.64	0.59
	lnc-CD46-4	-3.56	0.69
	lnc-CLGN-1	-3.48	0.75

Supplementary Table S2D: Modulated lncRNAs between MAF+ and MAF- MM patients.

	Gene Name	Score(d)	Fold Change
MAF +	lnc-ANKRD30B-7	3.58	5.78
	lnc-FAM65B-3	3.53	3.20
	lnc-CXorf36-1	3.28	3.81
	lnc-WDR89-1	3.20	1.44
	lnc-ACO1-6	3.18	2.32
	lnc-ZNF22-2	-6.17	0.28
	lnc-AC005493.1-1	-5.15	0.53
	lnc-MAP1LC3B2-2	-4.85	0.35
	lnc-MAP1LC3B2-4	-4.82	0.32
	lnc-MRPL39-5	-4.17	0.59
	lnc-OTX2-6	-3.94	0.57
	lnc-RXFP2-1	-3.89	0.50
	lnc-ANKS4B-1	-3.83	0.37
	lnc-JAM2-2	-3.76	0.08
	lnc-LIPG-3	-3.59	0.28
	lnc-FAM90A1-1	-3.55	0.59
	lnc-IER5-2	-3.49	0.54
	lnc-AC068020.1-1	-3.34	0.59
	lnc-C17orf46-3	-3.32	0.58
	lnc-CELA3A-1	-3.16	0.53
	lnc-FAM69C-2	-3.13	0.39
	lnc-RBM11-5	-3.10	0.66
	lnc-USP25-2	-3.06	0.38
	lnc-LTA4H-4	-3.05	0.66
	lnc-ZNF721-1	-2.98	0.52
	lnc-ARID5A-3	-2.94	0.53

Supplementary Table S3: List of 518 differentially expressed genes in MALAT quartile I vs quartile IV by SAM analysis at high stringency level (median FDR=0, 90th perc FDR=0). Genes are ordered according to SAM score, the fold change (FC) is also reported.

Gene Name	Score(d)	FC
ST20	6.9055	2.3966
NDUFAF3	6.6968	2.1369
CCDC97	6.6830	2.0026
MNF1	6.6295	2.6202
BTBD2	6.5947	1.8623
MRPS11	6.5381	2.5786
TMEM223	6.4864	2.2075
PEX11B	6.3185	2.1294
EXOSC4	6.3034	1.9055
COX5B	6.2897	2.9514
C9orf114	6.2499	1.5969
SNAPC3	6.2418	2.2073
ZNF641	6.2345	1.7449
PSMC4	6.2113	1.7165
MRPL51	6.1872	1.9746
SAMM50	6.1062	2.0815
MEA1	6.0647	2.1221
ORMDL2	5.9729	2.3305
ATP5G1	5.9625	4.4712
KCTD13	5.9300	1.8231
NABP2	5.9129	1.7890
GSK3A	5.8463	1.9480
HAUS1	5.7775	2.3305
C14orf1	5.7586	2.2388
TUBG1	5.6992	2.1535
MRPL40	5.6635	2.0639
MRPS14	5.6474	1.7811
MPI	5.6309	1.8136
SDHC	5.6045	1.9664
PTPLB	5.5957	1.8297
MLLT1	5.5652	1.6159
BRK1	5.5580	2.0752
HCFC1	5.5531	1.9860
C9orf123	5.5523	2.1626
C16orf70	5.5465	1.6159
DDT	5.5463	2.3499
NEDD8	5.5215	1.9167
TPGS2	5.5190	2.0950
UBL5	5.5026	1.6773
MPV17	5.4888	2.0583
C19orf47	5.4843	1.5391
DCAKD	5.4713	1.9583
LMAN2	5.4643	1.8887
PPP1R11	5.4640	1.7769
PPP1R7	5.4612	1.7142
SNAPIN	5.4521	1.6866
ZNRD1	5.4316	1.8471
POLR2H	5.4313	2.1925
NUDT5	5.4266	1.9715
MFF	5.4188	1.6968
MGC57346	5.3946	2.3561
C11orf24	5.3833	1.8428
NDUFV3	5.3810	1.7310
COX16	5.3789	2.0978
XRCC6BP1	5.3605	2.0809
PDK2	5.3585	1.6492
KIAA0195	5.3478	1.5550
NDRG3	5.3416	2.0911
MRPS24	5.3296	1.8271
NCDN	5.3021	1.7267
YAE1D1	5.2895	2.2750
GTF2A2	5.2885	2.2038

Gene Name	Score(d)	FC
MALAT1	-11.0931	-1.3961
USP47	-7.5270	-1.9633
SYF2	-7.3172	-2.1373
WDR48	-6.9632	-2.1348
GGNBP2	-6.8694	-2.0983
FXR1	-6.7628	-1.9732
MORF4L1	-6.7552	-1.7107
MFN1	-6.6958	-1.8638
EWSR1	-6.6385	-2.0545
DHX15	-6.6105	-1.8490
PNN	-6.5204	-1.9905
GTPBP4	-6.4839	-2.1545
SART1	-6.4743	-2.0592
MTMR14	-6.3970	-1.7561
PDCD4	-6.3798	-3.0419
NMD3	-6.3183	-2.3006
RNF6	-6.2967	-1.9764
KIN	-6.2831	-1.7434
RNF11	-6.2643	-1.9917
HNRNPM	-6.2301	-2.1023
HNRNPH1	-6.1444	-1.9047
NAA30	-6.1342	-1.7443
SREK1	-6.1031	-1.5718
DCUN1D1	-6.0888	-1.6630
GBE1	-6.0391	-1.9361
BNIP2	-6.0306	-1.7554
TMF1	-5.9383	-1.8334
HARBI1	-5.9191	-1.6589
RAB1A	-5.9012	-1.5449
RNF149	-5.8705	-1.8538
AMD1	-5.8648	-1.7263
B3GNT2	-5.8520	-2.3296
FAR1	-5.8506	-1.6563
INSIG2	-5.8222	-2.1010
SNX4	-5.7911	-1.9361
RABGEF1	-5.7645	-1.7396
SRSF4	-5.7583	-1.5838
ACAA1	-5.7427	-1.6193
RPSA	-5.7208	-3.0622
RABGGTB	-5.7110	-1.8517
SENP5	-5.6903	-1.6604
FKBP2	-5.6724	-2.9230
BTAF1	-5.6684	-1.5858
SRSF5	-5.6641	-1.8492
THRAP3	-5.6497	-1.5968
SNW1	-5.6422	-1.6881
PDIK1L	-5.6409	-2.0258
DNAJA2	-5.6256	-1.9115
C11orf58	-5.6235	-1.6583
DDX21	-5.6160	-1.8589
RAB18	-5.5982	-1.8403
SF3B2	-5.5975	-1.5471
FOXO4	-5.5797	-1.4455
RBM3	-5.5684	-1.6378
LEMD2	-5.5553	-1.4164
SPTY2D1	-5.5471	-1.8814
ISY1	-5.5379	-2.1647
CCNL1	-5.5280	-1.5936
AZIN1	-5.5261	-2.0774
DLD	-5.5165	-1.8402
ATAD1	-5.5001	-1.6658
CARS	-5.4866	-1.8002

Gene Name	Score(d)	FC
MED12	5.2790	1.7484
VAMP4	5.2618	1.9086
PRR12	5.2562	1.4400
XRCC1	5.2268	1.6764
TMEM141	5.2187	1.7945
PSMD8	5.2158	1.8141
SKP2	5.2125	2.0097
COMMD1	5.2113	1.5214
SPRYD4	5.1985	1.7956
TMEM14B	5.1911	1.8463
CLN6	5.1865	2.1009
SLC30A6	5.1725	1.7591
ATRAID	5.1656	1.9040
TMEM254	5.1610	2.1664
LOC100128288	5.1529	1.7704
COX6A1	5.1423	1.9900
PCGF6	5.1398	1.6136
CCDC167	5.1376	1.9311
LDB1	5.1266	1.6579
ATP6V1F	5.1199	1.9496
ARID1A	5.1123	1.8160
DENND4B	5.1107	1.6637
PHKG2	5.1034	1.5357
GSTM4	5.0946	2.0812
DCP1B	5.0806	1.8270
INTS3	5.0790	1.9138
ALG10B	5.0751	3.0766
TIMM50	5.0736	1.6016
TMEM179B	5.0566	1.9878
MRPS21	5.0544	2.1245
ACOT13	5.0481	2.4009
MRI1	5.0469	1.7706
HNRNPUL1	5.0424	1.5741
ASB12	5.0414	1.4162
UXT	5.0378	1.9093
STK38	5.0346	1.9371
TAF6	5.0307	1.5595
ZNF391	5.0273	2.7049
POLR2F	5.0269	1.4525
CLEC16A	5.0184	1.6075
CAPN3	5.0097	1.8714
ATXN2	5.0060	1.7640
TIMMDC1	5.0055	1.8774
SHKBP1	5.0035	1.5313
C11orf71	5.0012	1.7987
ATMIN	4.9998	1.6887
CIAO1	4.9928	1.4422
ZRANB3	4.9817	1.8413
C16orf13	4.9730	1.5260
PIGM	4.9673	1.7665
SH2D3C	4.9606	1.5953
C8orf40	4.9584	1.7562
MRPL43	4.9381	1.3519
ABI2	4.9303	1.8099
TMSB10	4.9260	1.8174
UFC1	4.9206	1.9551
LCLAT1	4.9192	1.9489
PAFAH1B3	4.9168	1.6509
STK25	4.9152	1.4148
CHAC2	4.9133	2.1256
ATP7A	4.9123	1.5664
PNPLA4	4.9108	2.0275

Gene Name	Score(d)	FC
ZFYVE20	-5.4826	-1.5610
ORMDL3	-5.4813	-1.9846
PRPF39	-5.4707	-1.6315
MINOS1	-5.4593	-2.5319
MED4	-5.4592	-1.7629
CLPX	-5.4312	-1.7572
DDX42	-5.4306	-1.8064
MAPRE1	-5.4300	-1.7543
GOLT1B	-5.4269	-2.2209
CASP7	-5.4263	-1.6764
PLEKHB2	-5.4197	-1.7010
MRFAP1	-5.3949	-1.7917
LUC7L3	-5.3847	-1.8373
MED17	-5.3728	-1.6832
CNBP	-5.3596	-1.9113
CXorf38	-5.3531	-1.4680
TARDBP	-5.3435	-1.6930
TRIM23	-5.3432	-1.8633
RBM17	-5.3215	-1.6782
CLK4	-5.3043	-1.6600
UIMC1	-5.2935	-1.5162
MIER1	-5.2743	-1.6661
TXNRD1	-5.2727	-1.8736
PARL	-5.2545	-1.8689
RBM39	-5.2496	-1.5650
TXN	-5.2450	-2.0488
GLUD1	-5.2340	-1.5647
RB1CC1	-5.2153	-1.9946
PSMD6	-5.2118	-1.5967
PITPNB	-5.2029	-1.9705
CYLD	-5.2003	-1.7966
RNF10	-5.1919	-1.4896
CCDC28A	-5.1862	-1.7492
SLU7	-5.1857	-1.8368
NAA38	-5.1801	-1.6866
ORAOV1	-5.1798	-1.4965
PSMD12	-5.1778	-1.5936
POU5F1P3	-5.1741	-1.9433
HAUS3	-5.1726	-2.1190
SPCS3	-5.1654	-1.6091
SAFB	-5.1570	-1.6475
PPM1B	-5.1509	-1.5250
GLRX3	-5.1140	-1.6352
BMS1	-5.1075	-1.5843
TOPORS	-5.1064	-1.6138
PI4K2B	-5.1058	-1.8679
FYTTD1	-5.0998	-1.7890
ZFAND6	-5.0933	-1.7441
ATP6V1H	-5.0816	-1.5558
MKRN1	-5.0812	-1.5131
HNRNPC	-5.0778	-1.5700
KDM1A	-5.0754	-1.4685
MARCKS	-5.0721	-2.1368
CNOT8	-5.0697	-1.5950
UBE2B	-5.0588	-1.6136
CREBZF	-5.0582	-1.7180
SAFB2	-5.0479	-1.7402
KTN1	-5.0461	-1.6044
PPP4R2	-5.0350	-1.7206
OPA1	-5.0348	-1.5936
KDM5A	-5.0299	-1.6019
PPP3CC	-5.0290	-1.7811

Gene Name	Score(d)	FC
MKL2	4.8973	1.6813
RAB3A	4.8947	1.6991
HDAC8	4.8890	1.6789
DNLZ	4.8874	1.9373
HAGH	4.8774	1.5566
BLZF1	4.8774	1.9892
CYB5A	4.8710	2.0824
NDUFA2	4.8701	1.7429
ZDHHC8	4.8601	1.4431
TAF4	4.8422	1.2712
DTYMK	4.8303	1.6624
SF3B5	4.8248	1.8477
PICK1	4.8229	1.4924
CHMP2A	4.8162	1.5064
NIT2	4.8159	1.9624
PNKP	4.8153	1.5211
NIF3L1	4.7847	1.8663
MBD6	4.7793	1.6033
POLR3K	4.7744	1.6640
NDUFA6	4.7704	1.7807
BCS1L	4.7676	1.5515
SNAP29	4.7659	1.7111
SCRN3	4.7615	1.6888
SRCAP	4.7602	1.6430
MRPL28	4.7509	1.6433
DGUOK	4.7501	1.5651
SH3BGRL	4.7437	1.5677
MYL12B	4.7336	2.1592
C12orf10	4.7271	1.6808
CDK4	4.7245	1.4722
RPAP1	4.7144	1.5257
ASB16-AS1	4.7133	1.7412
SELPLG	4.7133	2.6147
PDK3	4.7121	1.4636
HTRA2	4.7085	1.5368
ORAI3	4.6952	1.4636
STAM2	4.6870	1.5543
MALSU1	4.6863	1.7598
PFN1	4.6837	1.5370
PSMA2	4.6754	1.9985
STK36	4.6614	1.6248
RPAIN	4.6565	1.6984
TMEM258	4.6425	2.1021
PFDN6	4.6340	1.5668
APOO	4.6204	1.8674
KIAA1715	4.6202	1.5457
TP53TG1	4.6198	1.5869
ROMO1	4.6198	1.7767
ERAL1	4.6185	1.6175
OTUD6B	4.6182	1.6606
TRMT13	4.6166	1.8549
HDAC3	4.6164	1.4222
ATF1	4.6126	1.4529
EBAG9	4.6051	1.6779
CCDC53	4.6046	1.6861
TUBB	4.5946	1.5492
GDPGP1	4.5925	1.5417
TXNL4A	4.5846	1.6752
PSMD10	4.5831	1.7604
SIGMAR1	4.5727	2.1137
TMEM33	4.5578	1.3719
RPP38	4.5577	1.6305

Gene Name	Score(d)	FC
HBP1	-5.0288	-1.7914
RAB22A	-5.0214	-1.4676
BIN3	-5.0186	-1.6230
YTHDC1	-5.0155	-1.6278
DDX3X	-4.9887	-2.0073
RPL11	-4.9847	-1.7424
PCNP	-4.9813	-1.4256
DDX6	-4.9775	-1.6565
WBSCR16	-4.9639	-1.3674
SLMO2	-4.9566	-1.9295
SIRT7	-4.9500	-1.6467
ZNF451	-4.9434	-1.6280
TAX1BP1	-4.9422	-1.4908
BIRC2	-4.9392	-1.5386
NGLY1	-4.9333	-1.5754
ACBD5	-4.9275	-1.7223
RTN4	-4.9264	-1.4887
BAG5	-4.9243	-1.6044
SAMD8	-4.9201	-1.6180
HINT3	-4.8996	-1.7258
ARID4A	-4.8971	-1.8741
FAM76B	-4.8945	-1.3744
RSL24D1	-4.8757	-1.7242
CHD2	-4.8730	-1.6854
HNRNPA1	-4.8679	-1.5694
HNRNPA3P1	-4.8645	-1.3667
XPC	-4.8642	-1.5902
CHMP2B	-4.8593	-1.6474
METAP2	-4.8446	-1.5844
WDR82	-4.8407	-1.6093
PDCD10	-4.8397	-1.5397
ANXA7	-4.8390	-1.5821
FBXO11	-4.8384	-1.6052
RAD21	-4.8270	-1.7280
ZNF410	-4.8251	-1.6052
TAF7	-4.8191	-1.8926
LRFN4	-4.8141	-1.3734
CUL5	-4.8140	-1.4308
PPID	-4.8046	-1.4950
ARFGAP2	-4.7986	-1.5141
STRAP	-4.7925	-1.6082
PIK3CA	-4.7887	-1.6748
DHX38	-4.7864	-1.5011
PJA2	-4.7823	-1.5469
NUDT9	-4.7801	-1.8656
GMFB	-4.7781	-1.5689
CTSL1	-4.7699	-2.4912
NDUFA4	-4.7686	-1.7943
ATL2	-4.7641	-2.0313
MIB1	-4.7369	-1.8007
MYNN	-4.7332	-1.5083
HSBP1	-4.7209	-1.7552
SMARCA5	-4.7165	-1.6364
RAB21	-4.7162	-1.6423
PRPF4B	-4.7139	-1.5104
TCF24	-4.7126	-1.4748
OARD1	-4.7110	-1.6478
LSM14A	-4.7030	-1.5119
EMC6	-4.7005	-1.8160
TRAM1	-4.6987	-1.5575
APOL2	-4.6943	-1.7544
ARMCX3	-4.6854	-1.7585

Gene Name	Score(d)	FC
CABLES2	4.5529	1.4763
MLX	4.5440	1.5122
AP2A1	4.5336	1.5652
MAP4K1	4.5252	1.4991
MRGBP	4.5199	1.3983
RAB5C	4.5183	1.7957
ASXL2	4.5125	1.9191
R3HDM1	4.5059	1.5788
STK3	4.4979	1.5247
MYD88	4.4914	1.8876
ZNF717	4.4842	1.8085
TBC1D19	4.4763	1.6893
ARHGEF9	4.4762	1.5943
ATP6V0E1	4.4757	1.6175
RNLS	4.4729	1.3770
TCF12	4.4728	1.7134
ATF7	4.4700	1.5130
TULP3	4.4688	1.7455
C15orf41	4.4587	1.8601
POGZ	4.4575	1.6289
ANO6	4.4499	1.6435
PDE6D	4.4485	1.4982
FAM217B	4.4433	1.6728
MRPS18A	4.4336	1.7542
ZBTB8OS	4.4250	2.3646
ZC3H4	4.4222	1.2936
CCDC92	4.4215	1.6799
TUBG2	4.4176	1.7653
ASB8	4.4143	1.6252
EXOSC1	4.4060	1.4852
ZNF384	4.4051	1.5012
NDUFA12	4.4000	1.5524
PIP5K1C	4.3957	1.4412
PSENEN	4.3912	1.8941
VMAC	4.3908	1.3894
RNF26	4.3894	1.6276
DTD2	4.3872	1.6407
ZNF138	4.3838	1.7540
GGA3	4.3788	1.4538
MED31	4.3761	1.4883
EXOC7	4.3708	1.3703
YWHAQ	4.3689	1.4622
SLC25A40	4.3689	1.7418

Gene Name	Score(d)	FC
CSNK1A1	-4.6837	-1.5345
USP48	-4.6780	-1.5535
RNF113A	-4.6763	-1.5099
RECQL	-4.6704	-1.8005
CBX3	-4.6694	-1.4652
PSMC3	-4.6647	-1.5788
RAB27A	-4.6570	-2.0908
RP9	-4.6511	-1.5602
CDC37L1	-4.6453	-1.6749
PMS2P3	-4.6420	-1.4218
TAPT1	-4.6368	-1.5947
PPP1R3B	-4.6324	-1.5690
CLP1	-4.6299	-1.5687
GSKIP	-4.6257	-1.6375
IER5	-4.6216	-1.8026
YKT6	-4.6129	-1.4592
CCDC12	-4.6076	-1.5873
MRPL22	-4.6062	-1.6375
EIF5B	-4.6016	-1.4147
RALB	-4.6006	-1.5959
CCDC126	-4.5970	-1.6440
EIF4A2	-4.5966	-1.6035
PRKRA	-4.5909	-1.4846
ARPC3	-4.5894	-1.5133
PDIA6	-4.5868	-1.8216
PPP3R1	-4.5798	-1.6198
MTMR6	-4.5731	-1.6316
CDK5RAP3	-4.5715	-1.9961
SUMO1	-4.5705	-1.5715
MPP5	-4.5636	-1.6337
PNISR	-4.5613	-1.4394
RNMT	-4.5582	-1.6034
AAGAB	-4.5578	-1.7048
EIF3A	-4.5574	-1.3204
FBXL3	-4.5544	-1.7251
SMIM15	-4.5433	-1.6124
TPGS1	-4.5324	-1.6815
UBA5	-4.5251	-1.5451
NDUFA5	-4.5235	-2.1310
BRMS1L	-4.5220	-1.9349
SLC36A4	-4.5197	-1.5858
SLC35A3	-4.5124	-1.7416
GALNT4	-4.5049	-1.5147
SLC39A7	-4.5046	-1.7145
NCAPH2	-4.5018	-1.3248
GRSF1	-4.4952	-1.5520
MIR494	-4.4931	-1.4970
OSBPL2	-4.4927	-1.4020
NAP1L1	-4.4842	-1.6252
NANP	-4.4832	-1.4779
FGFR1OP2	-4.4759	-1.6896
C1orf63	-4.4747	-1.7449
TFAM	-4.4714	-1.8069
OPTN	-4.4712	-1.7482
PRPF38B	-4.4672	-1.4626
SEC61G	-4.4595	-1.4982
NOC2L	-4.4591	-1.4791
NFYB	-4.4590	-2.1508
RNU6-57	-4.4546	-1.3757
GLOD4	-4.4515	-2.3187
RIT1	-4.4493	-1.6956
MRPS10	-4.4450	-1.5753

Gene Name	Score(d)	FC
**CWF19L1**	-4.4446	-1.4883
**RANBP6**	-4.4445	-1.5874
**EEF1A1**	-4.4412	-2.2670
**MIR149**	-4.4342	-1.4636
**POLR2B**	-4.4309	-1.5823
**SENP2**	-4.4254	-1.4917
**VPS18**	-4.4235	-1.3367
**SART3**	-4.4235	-1.6957
**SLC25A26**	-4.4233	-1.5259
**ZNRF2**	-4.4218	-1.3841
**TSR2**	-4.4202	-1.6452
**RPL6**	-4.4189	-1.3340
**SOD2**	-4.4062	-1.8681
**RAB2A**	-4.3897	-1.4638
**ATG14**	-4.3897	-1.8524
**C1QL1**	-4.3861	-1.3152
**USP33**	-4.3796	-1.6350
**TSPAN3**	-4.3794	-1.6692
**MATR3**	-4.3780	-1.3836
**ZBTB34**	-4.3778	-1.4426
**HIAT1**	-4.3774	-1.7750
**ZNF622**	-4.3748	-1.6658
**TOMM70A**	-4.3715	-1.5352
**LOC400657**	-4.3702	-1.9517
**RCN1**	-4.3648	-2.4491
**RBAK**	-4.3603	-1.5540
**FBXO34**	-4.3585	-1.5315
**LMNB1**	-4.3583	-1.8352
**KRTAP19-5**	-4.3571	-1.4593
**CRLF3**	-4.3528	-1.9139
**ZUFSP**	-4.3348	-1.6791
**BCLAF1**	-4.3347	-1.4785
**PEX16**	-4.3304	-1.2884
**NAP1L4**	-4.3276	-1.4298
**PNRC1**	-4.3276	-1.4043
**RLIM**	-4.3264	-1.3939
**VEZT**	-4.3141	-1.4829
**MYCBP**	-4.3129	-1.4099
**TBK1**	-4.3120	-1.4319
**FTSJD2**	-4.3117	-1.4923
**ADAM17**	-4.3047	-1.5215

Supplementary Table S4. List of the significantly enriched pathways for the 518 differentially expressed genes, by Toppgene suite analysis.

Category	Source	ID	Name	q-value FDR B&H	Hit Count in Query List	Hit Count in Genome	Hit in Query List
Pathway	BioSystems: KEGG	125136	Spliceosome	5.48E-07	20	131	DDX42,HNRNPA1,HNRNPC,ISY1,SRSF4,SRSF5,SLU7,SYF2,CCDC12,SNW1,LSM8,SF3B5,DHX38,HNRNPM,DHX15,TXNL4A,SF3B2,PRPF38B,SART1,RBM17
Pathway	BioSystems: WikiPathways	198843	mRNA processing	3.51E-06	19	136	CLK4,HNRNPA1,HNRNPC,HNRNPH1,SRSF4,SRSF5,RBM39,SF3B5,RNMT,DHX38,HNRNPM,SREK1,DHX15,TXNL4A,PRPF4B,CLP1,SF3B2,HNRNPA3P1,RBM17
Pathway	BioSystems: REACTOME	105952	mRNA Splicing - Major Pathway	1.31E-04	15	112	HNRNPA1,HNRNPC,HNRNPH1,SRSF4,SRSF5,POLR2B,POLR2F,POLR2H,SF3B5,DHX38,HNRNPM,TXNL4A,CLP1,SF3B2,HNRNPUL1
Pathway	BioSystems: REACTOME	105951	mRNA Splicing	1.31E-04	15	112	HNRNPA1,HNRNPC,HNRNPH1,SRSF4,SRSF5,POLR2B,POLR2F,POLR2H,SF3B5,DHX38,HNRNPM,TXNL4A,CLP1,SF3B2,HNRNPUL1
Pathway	BioSystems: REACTOME	160950	Processing of Capped Intron-Containing Pre-mRNA	2.36E-03	15	143	HNRNPA1,HNRNPC,HNRNPH1,SRSF4,SRSF5,POLR2B,POLR2F,POLR2H,SF3B5,DHX38,HNRNPM,TXNL4A,CLP1,SF3B2,HNRNPUL1
Pathway	BioSystems: KEGG	83100	Huntington's disease	2.57E-03	17	183	AP2A1,SDHC,COX5B,POLR2B,COX6A1,POLR2F,POLR2H,SOD2,ATP5G1,NDUFA2,NDUFA4,NDUFA5,NDUFA6,NDUFV3,NDUFA12,TAF4,TFAM
Pathway	BioSystems: REACTOME	105983	HIV Infection	2.68E-03	19	225	AP2A1,POLR2B,POLR2F,POLR2H,CHMP2B,ATP6V1H,RNMT,PSMA2,PSMC3,PSMC4,PSMD8,PSMD10,PSMD12,CHMP2A,PSMD6,TAF4,TAF6,CUL5,GTF2A2
Pathway	BioSystems: REACTOME	106176	Regulation of ornithine decarboxylase (ODC)	8.22E-03	8	50	AZIN1,PSMA2,PSMC3,PSMC4,PSMD8,PSMD10,PSMD12,PSMD6
Pathway	BioSystems: KEGG	83097	Alzheimer's disease	8.22E-03	15	168	SDHC,COX5B,COX6A1,PPP3CC,PPP3R1,ATP5G1,PSENEN,NDUFA2,NDUFA4,NDUFA5,NDUFA6,NDUFV3,NDUFA12,ADAM17,CASP7
Pathway	BioSystems: REACTOME	105796	Ubiquitin-dependent degradation of Cyclin D	8.22E-03	8	51	PSMA2,PSMC3,PSMC4,PSMD8,PSMD10,PSMD12,PSMD6,CDK4
Pathway	BioSystems: REACTOME	105797	Ubiquitin-dependent degradation of Cyclin D1	8.22E-03	8	51	PSMA2,PSMC3,PSMC4,PSMD8,PSMD10,PSMD12,PSMD6,CDK4
Pathway	BioSystems: KEGG	82942	Oxidative phosphorylation	8.22E-03	13	133	ATP6V1F,SDHC,COX5B,COX6A1,ATP6V1H,ATP5G1,NDUFA2,NDUFA4,NDUFA5,NDUFA6,NDUFV3,NDUFA12,ATP6V0E1
Pathway	BioSystems: REACTOME	477137	The citric acid (TCA) cycle and respiratory electron transport	8.88E-03	13	136	PDK2,PDK3,SDHC,COX5B,COX6A1,ATP5G1,NDUFA2,NDUFA4,NDUFA5,NDUFA6,NDUFV3,NDUFA12,DLD
Pathway	MSigDB C2: BioCarta	M194	Proteasome Complex	8.88E-03	6	28	PSMA2,PSMC3,PSMC4,PSMD8,PSMD12,PSMD6
Pathway	BioSystems: WikiPathways	198917	Eukaryotic Transcription Initiation	9.96E-03	7	41	POLR2B,POLR2F,POLR2H,POLR3K,TAF6,TAF7,GTF2A2
Pathway	BioSystems: REACTOME	105937	Gene Expression	9.98E-03	54	1188	CBX3,HNRNPA1,HNRNPC,HNRNPH1,CNOT8,SPCS3,MED17,EXOSC4,SRSF4,SRSF5,POLR2B,POLR2F,POLR2H,PRKRA,MED4,SNW1,ZNF641,EIF5B,SNAPC3,SF3B5,ZNF717,POLR3K,ZNF138,RNMT,PSMA2,DHX38,HNRNPM,PSMC3,PSMC4,PSMD8,PSMD10,PSMD12,DDX6,PSMD6,TXNL4A,TAF4,TAF6,CLP1,MED12,SF3B2,MED31,CARS,EXOSC1,RPSA,HNRNPUL1,TFAM,EEF1A1,GTF2A2,DCP1B,TRAM1,EIF4A2,SEC61G,RPL6,RPL11
Pathway	BioSystems: REACTOME	106557	RNA Polymerase II Transcription	9.98E-03	11	104	SRSF4,SRSF5,POLR2B,POLR2F,POLR2H,RNMT,DHX38,TAF4,TAF6,CLP1,GTF2A2
Pathway	BioSystems: REACTOME	106017	Vif-mediated degradation of APOBEC3G	9.98E-03	8	56	PSMA2,PSMC3,PSMC4,PSMD8,PSMD10,PSMD12,PSMD6,CUL5
Pathway	BioSystems: REACTOME	105648	Apoptosis	1.02E-02	14	162	BIRC2,PPP3CC,PPP3R1,PSMA2,PSMC3,PSMC4,PSMD8,PSMD10,PSMD12,PSMD6,ADAM17,YWHAQ,CASP7,LMNB1
Pathway	BioSystems: REACTOME	105771	SCF(Skp2)-mediated degradation of p27/p21	1.02E-02	8	57	SKP2,PSMA2,PSMC3,PSMC4,PSMD8,PSMD10,PSMD12,PSMD6
Pathway	GenMAPP	MAP00190_Oxidative_phosphorylation	MAP00190 Oxidative phosphorylation	1.13E-02	7	44	ATP6V1F,COX5B,COX6A1,ATP7A,NDUFA5,ATP6V0E1,MPP5
Pathway	PantherDB	P00023	General transcription regulation	1.24E-02	6	32	POLR2F,POLR2H,TAF4,TAF6,TAF7,GTF2A2

Category	Source	ID	Name	q-value FDR B&H	Hit Count in Query List	Hit Count in Genome	Hit in Query List
Pathway	MSigDB C2: BioCarta	M8179	Rab GTPases Mark Targets In The Endocytotic Machinery	1.35E-02	4	12	RAB1A,RAB2A,RAB3A,RAB27A
Pathway	BioSystems: KEGG	862188	Non-alcoholic fatty liver disease (NAFLD)	1.48E-02	13	151	PIK3CA,SDHC,COX5B,COX6A1,NDUFA2,NDUFA4,NDUFA5,NDUFA6,NDUFV3,NDUFA12,MLX,CASP7,GSK3A
Pathway	BioSystems: REACTOME	160962	Respiratory electron transport	1.56E-02	9	79	SDHC,COX5B,COX6A1,NDUFA2,NDUFA4,NDUFA5,NDUFA6,NDUFV3,NDUFA12
Pathway	BioSystems: REACTOME	105921	Respiratory electron transport, ATP synthesis by chemiosmotic coupling, and heat production by uncoupling proteins.	1.56E-02	10	97	SDHC,COX5B,COX6A1,ATP5G1,NDUFA2,NDUFA4,NDUFA5,NDUFA6,NDUFV3,NDUFA12
Pathway	BioSystems: WikiPathways	198884	TNF-alpha/NF-kB Signaling Pathway	1.56E-02	15	196	COMMD1,SUMO1,POLR2H,BIRC2,TBK1,CYLD,PSMC3,PSMD12,DDX3X,PSMD6,YWHAQ,KTN1,CAPN3,CASP7,RPL6
Pathway	BioSystems: REACTOME	366161	Class I MHC mediated antigen processing & presentation	1.56E-02	18	263	UBA5,MKRN1,UBE2B,ASB8,ASB12,SKP2,CTSL,PSMA2,PSMC3,PSMC4,PSMD8,PSMD10,PSMD12,PSMD6,PJA2,CUL5,RNF6,SEC61G
Pathway	BioSystems: REACTOME	477126	Cross-presentation of soluble exogenous antigens (endosomes)	1.56E-02	7	49	PSMA2,PSMC3,PSMC4,PSMD8,PSMD10,PSMD12,PSMD6
Pathway	BioSystems: REACTOME	477122	Antigen processing-Cross presentation	1.56E-02	9	81	CTSL,PSMA2,PSMC3,PSMC4,PSMD8,PSMD10,PSMD12,PSMD6,SEC61G
Pathway	BioSystems: REACTOME	366162	Antigen processing: Ubiquitination & Proteasome degradation	1.56E-02	16	220	UBA5,MKRN1,UBE2B,ASB8,ASB12,SKP2,PSMA2,PSMC3,PSMC4,PSMD8,PSMD10,PSMD12,PSMD6,PJA2,CUL5,RNF6
Pathway	BioSystems: REACTOME	477124	ER-Phagosome pathway	1.56E-02	8	65	PSMA2,PSMC3,PSMC4,PSMD8,PSMD10,PSMD12,PSMD6,SEC61G
Pathway	BioSystems: WikiPathways	198786	Proteasome Degradation	1.56E-02	8	65	UBE2B,PSMA2,PSMC3,PSMC4,PSMD8,PSMD10,PSMD12,PSMD6
Pathway	BioSystems: REACTOME	105686	Regulation of activated PAK-2p34 by proteasome mediated degradation	1.58E-02	7	50	PSMA2,PSMC3,PSMC4,PSMD8,PSMD10,PSMD12,PSMD6
Pathway	BioSystems: REACTOME	105770	Cyclin E associated events during G1/S transition	1.64E-02	8	66	SKP2,PSMA2,PSMC3,PSMC4,PSMD8,PSMD10,PSMD12,PSMD6
Pathway	BioSystems: REACTOME	105788	CDK-mediated phosphorylation and removal of Cdc6	1.69E-02	7	51	PSMA2,PSMC3,PSMC4,PSMD8,PSMD10,PSMD12,PSMD6
Pathway	BioSystems: REACTOME	105783	Cyclin A:Cdk2-associated events at S phase entry	1.70E-02	8	67	SKP2,PSMA2,PSMC3,PSMC4,PSMD8,PSMD10,PSMD12,PSMD6
Pathway	BioSystems: REACTOME	106026	Vpu mediated degradation of CD4	1.70E-02	7	52	PSMA2,PSMC3,PSMC4,PSMD8,PSMD10,PSMD12,PSMD6
Pathway	BioSystems: REACTOME	105821	Assembly of the pre-replicative complex	1.70E-02	8	68	MCM2,PSMA2,PSMC3,PSMC4,PSMD8,PSMD10,PSMD12,PSMD6
Pathway	BioSystems: REACTOME	105748	p53-Independent DNA Damage Response	1.70E-02	7	53	PSMA2,PSMC3,PSMC4,PSMD8,PSMD10,PSMD12,PSMD6
Pathway	BioSystems: REACTOME	160939	Autodegradation of the E3 ubiquitin ligase COP1	1.70E-02	7	53	PSMA2,PSMC3,PSMC4,PSMD8,PSMD10,PSMD12,PSMD6
Pathway	BioSystems: REACTOME	105747	p53-Independent G1/S DNA damage checkpoint	1.70E-02	7	53	PSMA2,PSMC3,PSMC4,PSMD8,PSMD10,PSMD12,PSMD6
Pathway	BioSystems: REACTOME	105749	Ubiquitin Mediated Degradation of Phosphorylated Cdc25A	1.70E-02	7	53	PSMA2,PSMC3,PSMC4,PSMD8,PSMD10,PSMD12,PSMD6
Pathway	BioSystems: REACTOME	106511	Degradation of beta-catenin by the destruction complex	1.70E-02	8	69	CSNK1A1,PSMA2,PSMC3,PSMC4,PSMD8,PSMD10,PSMD12,PSMD6
Pathway	BioSystems: REACTOME	105782	S Phase	1.70E-02	11	124	MCM2,SKP2,PSMA2,PSMC3,PSMC4,PSMD8,PSMD10,PSMD12,PSMD6,RAD21,CDK4
Pathway	BioSystems: REACTOME	105744	Stabilization of p53	1.70E-02	7	54	PSMA2,PSMC3,PSMC4,PSMD8,PSMD10,PSMD12,PSMD6
Pathway	BioSystems: WikiPathways	198860	Electron Transport Chain	1.70E-02	10	106	SDHC,COX5B,COX6A1,ATP5G1,NDUFA2,NDUFA4,NDUFA5,NDUFA6,NDUFV3,NDUFA12
Pathway	BioSystems: REACTOME	105786	Switching of origins to a post-replicative state	1.70E-02	8	71	MCM2,PSMA2,PSMC3,PSMC4,PSMD8,PSMD10,PSMD12,PSMD6
Pathway	BioSystems: REACTOME	105787	Orc1 removal from chromatin	1.70E-02	8	71	MCM2,PSMA2,PSMC3,PSMC4,PSMD8,PSMD10,PSMD12,PSMD6
Pathway	BioSystems: REACTOME	105942	RNA Polymerase II Transcription Initiation And Promoter Clearance	1.70E-02	6	40	POLR2B,POLR2F,POLR2H,TAF4,TAF6,GTF2A2
Pathway	BioSystems: REACTOME	105999	RNA Polymerase II HIV Promoter Escape	1.70E-02	6	40	POLR2B,POLR2F,POLR2H,TAF4,TAF6,GTF2A2
Pathway	BioSystems: REACTOME	105998	HIV Transcription Initiation	1.70E-02	6	40	POLR2B,POLR2F,POLR2H,TAF4,TAF6,GTF2A2
Pathway	BioSystems: REACTOME	105944	RNA Polymerase II Promoter Escape	1.70E-02	6	40	POLR2B,POLR2F,POLR2H,TAF4,TAF6,GTF2A2
Pathway	BioSystems: REACTOME	105941	RNA Polymerase II Transcription Pre-Initiation And Promoter Opening	1.70E-02	6	40	POLR2B,POLR2F,POLR2H,TAF4,TAF6,GTF2A2
Pathway	BioSystems: REACTOME	105943	RNA Polymerase II Transcription Initiation	1.70E-02	6	40	POLR2B,POLR2F,POLR2H,TAF4,TAF6,GTF2A2
Pathway	BioSystems: REACTOME	187212	Destabilization of mRNA by AUF1 (hnRNP D0)	1.70E-02	7	55	PSMA2,PSMC3,PSMC4,PSMD8,PSMD10,PSMD12,PSMD6
Pathway	BioSystems: REACTOME	105828	SCF-beta-TrCP mediated degradation of Emi1	1.70E-02	7	55	PSMA2,PSMC3,PSMC4,PSMD8,PSMD10,PSMD12,PSMD6
Pathway	BioSystems: REACTOME	187211	Regulation of mRNA Stability by Proteins that Bind AU-rich Elements	1.70E-02	9	89	EXOSC4,PSMA2,PSMC3,PSMC4,PSMD8,PSMD10,PSMD12,PSMD6,EXOSC1
Pathway	BioSystems: REACTOME	105835	APC/C:Cdh1 mediated degradation of Cdc20 and other APC/C:Cdh1 targeted proteins in late mitosis/early G1	1.89E-02	8	73	SKP2,PSMA2,PSMC3,PSMC4,PSMD8,PSMD10,PSMD12,PSMD6
Pathway	BioSystems: REACTOME	105901	Removal of licensing factors from origins	1.89E-02	8	73	MCM2,PSMA2,PSMC3,PSMC4,PSMD8,PSMD10,PSMD12,PSMD6

Category	Source	ID	Name	q-value FDR B&H	Hit Count in Query List	Hit Count in Genome	Hit in Query List
Pathway	BioSystems: KEGG	83098	Parkinson's disease	2.11E-02	11	131	SDHC,COX5B,COX6A1,ATP5G1,NDUFA2,NDUFA4,NDUFA5,NDUFA6,NDUFV3,NDUFA12,HTRA2
Pathway	BioSystems: REACTOME	105953	mRNA Splicing - Minor Pathway	2.25E-02	6	43	POLR2B,POLR2F,POLR2H,SF3B5,TXNL4A,SF3B2
Pathway	BioSystems: REACTOME	105899	Regulation of DNA replication	2.25E-02	8	76	MCM2,PSMA2,PSMC3,PSMC4,PSMD8,PSMD10,PSMD12,PSMD6
Pathway	BioSystems: REACTOME	105743	p53-Dependent G1 DNA Damage Response	2.25E-02	7	59	PSMA2,PSMC3,PSMC4,PSMD8,PSMD10,PSMD12,PSMD6
Pathway	BioSystems: REACTOME	105741	p53-Dependent G1/S DNA damage checkpoint	2.25E-02	7	59	PSMA2,PSMC3,PSMC4,PSMD8,PSMD10,PSMD12,PSMD6
Pathway	BioSystems: REACTOME	105824	CDT1 association with the CDC6:ORC:origin complex	2.25E-02	7	59	PSMA2,PSMC3,PSMC4,PSMD8,PSMD10,PSMD12,PSMD6
Pathway	BioSystems: KEGG	83040	Proteasome	2.40E-02	6	44	PSMA2,PSMC3,PSMC4,PSMD8,PSMD12,PSMD6
Pathway	BioSystems: KEGG	83036	Ribosome	2.42E-02	11	135	MRPS21,MRPL28,MRPL22,MRPS14,MRPS11,RPSA,MRPS18A,MRPS10,RPL6,RSL24D1,RPL11
Pathway	BioSystems: REACTOME	105685	Regulation of Apoptosis	2.63E-02	7	61	PSMA2,PSMC3,PSMC4,PSMD8,PSMD10,PSMD12,PSMD6
Pathway	BioSystems: KEGG	585562	Epstein-Barr virus infection	2.77E-02	14	203	PIK3CA,POLR2B,POLR2F,POLR2H,SKP2,SNW1,TBK1,POLR3K,PSMC3,PSMC4,PSMD8,PSMD12,PSMD6,YWHAQ
Pathway	BioSystems: REACTOME	105740	G1/S DNA Damage Checkpoints	2.77E-02	7	62	PSMA2,PSMC3,PSMC4,PSMD8,PSMD10,PSMD12,PSMD6
Pathway	BioSystems: REACTOME	105996	Transcription of the HIV genome	2.77E-02	7	62	POLR2B,POLR2F,POLR2H,RNMT,TAF4,TAF6,GTF2A2
Pathway	Pathway Ontology	PW:0000315	calcineurin signaling	2.79E-02	3	9	PPP3CC,PPP3R1,RCAN1
Pathway	BioSystems: KEGG	83037	RNA polymerase	3.00E-02	5	32	ZNRD1,POLR2B,POLR2F,POLR2H,POLR3K
Pathway	PantherDB	P00055	Transcription regulation by bZIP transcription factor	3.05E-02	6	47	POLR2F,POLR2H,TAF4,TAF6,TAF7,GTF2A2
Pathway	BioSystems: REACTOME	105836	Autodegradation of Cdh1 by Cdh1:APC/C	3.17E-02	7	64	PSMA2,PSMC3,PSMC4,PSMD8,PSMD10,PSMD12,PSMD6
Pathway	BioSystems: REACTOME	105812	M Phase	3.28E-02	15	232	LEMD2,BLZF1,MAPRE1,PSMA2,HDAC8,PSMC3,PSMC4,PSMD8,PSMD10,PSMD12,PSMD6,RAB1A,RAB2A,RAD21,LMNB1
Pathway	BioSystems: REACTOME	645336	Synthesis of PIPs at the plasma membrane	3.28E-02	5	33	PI4K2B,PIK3CA,PIP5K1C,MTMR6,MTMR14
Pathway	BioSystems: REACTOME	105898	DNA Replication Pre-Initiation	3.55E-02	8	84	MCM2,PSMA2,PSMC3,PSMC4,PSMD8,PSMD10,PSMD12,PSMD6
Pathway	BioSystems: REACTOME	105820	M/G1 Transition	3.55E-02	8	84	MCM2,PSMA2,PSMC3,PSMC4,PSMD8,PSMD10,PSMD12,PSMD6
Pathway	BioSystems: REACTOME	160993	Deadenylation-dependent mRNA decay	3.90E-02	6	50	CNOT8,EXOSC4,DDX6,EXOSC1,DCP1B,EIF4A2
Pathway	BioSystems: REACTOME	160944	Regulation of mitotic cell cycle	3.94E-02	8	86	SKP2,PSMA2,PSMC3,PSMC4,PSMD8,PSMD10,PSMD12,PSMD6
Pathway	BioSystems: REACTOME	105825	APC/C-mediated degradation of cell cycle proteins	3.94E-02	8	86	SKP2,PSMA2,PSMC3,PSMC4,PSMD8,PSMD10,PSMD12,PSMD6
Pathway	BioSystems: REACTOME	106230	Metabolism of proteins	3.94E-02	32	689	TUBB2A,SPCS3,SUMO1,SAMM50,PFDN6,EXOSC4,MPI,SLC30A6,B3GNT2,TIMM50,EIF5B,GALNT4,ATP5G1,BCS1L,FBXL3,TOMM70A,NFYB,EXOC7,RAB27A,SENP5,EXOSC1,RPSA,GSK3A,ALG10B,EEF1A1,PDIA6,TRAM1,EIF4A2,SEC61G,RPL6,RPL11,PIGM
Pathway	BioSystems: REACTOME	576251	Activation of NF-kappaB in B Cells	3.98E-02	7	68	PSMA2,PSMC3,PSMC4,PSMD8,PSMD10,PSMD12,PSMD6
Pathway	BioSystems: REACTOME	105833	APC/C:Cdc20 mediated degradation of Securin	3.98E-02	7	68	PSMA2,PSMC3,PSMC4,PSMD8,PSMD10,PSMD12,PSMD6
Pathway	BioSystems: REACTOME	645332	PI Metabolism	4.02E-02	6	51	PI4K2B,PIK3CA,PITPNB,PIP5K1C,MTMR6,MTMR14
Pathway	BioSystems: REACTOME	106335	MicroRNA (miRNA) Biogenesis	4.98E-02	4	23	POLR2B,POLR2F,POLR2H,PRKRA

Supplementary Table S5. Selected gene sets significantly up- and down-regulated in MALAT1 quartile IV versus I by GSEA analysis. Genes contributing to the core enrichment in each gene set are indicated in bold.

Gene set name	NES	Gene set size	Gene list
REACTOME_CASPASE_MEDIATED_CLEAVAGE_OF_CYTOSKELETAL_PROTEINS	1.8397	11	**CASP7, GSN, CASP3, ADD1, CASP6**, SPTAN1, CASP8, VIM, DBNL, MAPT, GAS2
BIOPOLYMER_BIOSYNTHETIC_PROCESS	1.7961	10	**EEF1A1, TSFM, TUFM, GYG2**, B3GNT8, ALG1, DYRK2, GCK, EEF2K, GYS2
ATP_DEPENDENT_DNA_HELICASE_ACTIVITY	1.7101	10	**RECQL, CHD2, CHD1, DHX9, RUVBL2, XRCC6, CHD4**, XRCC5, PIF1, CHD3
SMOOTH_MUSCLE_CONTRACTION_GO_0006939	1.7078	12	**SPHK1, SOD1, NMU**, PLCE1, ADRA1A, KCNB2, SMTN, NMUR1, KNG1, CNN1, PROK2, MYH11
BOYAULT_LIVER_CANCER_SUBCLASS_G6_DN	1.6905	18	**CDH1, RRBP1, SYT17, SDC4, CMTM6, GATA6, AGO2**, TRIM15, OSBPL9, LDLR, HES1, INPP1, SLC20A1, LSR, NUP98, TNS1, RFTN1, TNFSF10
KOBAYASHI_EGFR_SIGNALING_6HR_UP	1.5956	7	**PDCD4, CCNG2, KLHL24, PELI1**, METTL7A, CEP68, AQP3
DRUG_BINDING	1.5939	15	**FKBP2, FKBP3, PPID, PPIH, PPIE, PPIG, PPIC**, ACR, NKTR, FKBP5, FKBP6, ALB, NFATC1, FKBP4, TOP2A
KERLEY_RESPONSE_TO_CISPLATIN_DN	1.5391	5	**MYC, HESX1, CYP26A1, HMGCS1, LPIN1**
ATP_DEPENDENT_HELICASE_ACTIVITY	-1.7369	24	**RECQL, DDX3X, DDX21, DDX43, DHX38, CHD2, CHD1, DHX9, DHX8, DDX18, RUVBL2, XRCC6, CHD4, UPF1, DDX25**, DDX54, XRCC5, DDX20, PIF1, CHD3, SKIV2L, DDX23, DDX56, EIF4A3
REACTOME_NOREPINEPHRINE_NEUROTRANSMITTER_RELEASE_CYCLE	-1.6592	12	**VAMP2, SYT1, RAB3A, STXBP1**, CPLX1, STX1A, DBH, SNAP25, SLC22A2, MAOA, SLC18A2, RIMS1
NADERI_BREAST_CANCER_PROGNOSIS_DN	-1.6542	16	**CLK4, REXO2, TGFBR3, C1S, DCN, MCEE, SHOX2, SPARCL1, TXNIP, ANXA5, FBLN1**, CLDN1, SMOC2, CEBPD, OMD, SSR2
REACTOME_DOPAMINE_NEUROTRANSMITTER_RELEASE_CYCLE	-1.6171	14	**VAMP2, SYT1, RAB3A, STXBP1**, CPLX1, STX1A, DDC, SYN1, SYN3, TH, SNAP25, SYN2, SLC18A2, RIMS1
DNA_DAMAGE_RESPONSESIGNAL_TRANSDUCTION_RESULTING_IN_INDUCTION_OF_APOPTOSIS	-1.5768	13	**CIDEB, TP53, BCL3, PCBP4, BRCA1, IFI16, ABL1, PML**, DYRK2, SFN, CIDEA, TP73, CHEK2

Supplementary Table S6: Sequence-specific SYBR green qRT-PCR primers

Primer used for Sybr-green qRT-PCR	
lnc-ANGPTL1-3_F	GCGTTTCTTCCAAATCACCT
lnc-ANGPTL1-3_R	GGAACCGGGCTCTAGAAAAA
lnc-SENP5-4_F	AAGCCAGAGAGCCTTTCCTC
lnc-SENP5-4_R	ATTTGGCAGCTGTAGGGTTG
lnc-LRRC47-1_F	CCGAGATGCTCTCGTGACTT
lnc-LRRC47-1_R	CTCAGCTCTGAAGGCTTGCT
